# Infinitesimal homeostasis in mass-action systems

**DOI:** 10.1007/s00285-026-02352-y

**Published:** 2026-02-17

**Authors:** Jiaxin Jin, Grzegorz A. Rempala

**Affiliations:** 1 Department of Mathematics, University of Louisiana at Lafayette, Lafayette, LA 70504 USA; 2 Department of Mathematics, The Ohio State University, Columbus, OH 43210 USA; 3 Division of Biostatistics, The Ohio State University, Columbus, OH 43210 USA

**Keywords:** Homeostasis, Chemical Reaction Network, Input-Output Network, Biochemistry, 34C99, 92C42, 92C40, 92C45

## Abstract

Homeostasis occurs in a biological system when a chosen output variable remains approximately constant despite changes in an input variable. In this work we specifically focus on biological systems that may be represented as chemical reaction networks and consider their infinitesimal homeostasis, where the derivative of the input-output function is zero. The specific challenge of chemical reaction networks is that they often obey various conservation laws complicating the standard input-output analysis. We derive several results that allow us to verify the existence of infinitesimal homeostasis points both in the absence of conservation and under conservation laws where conserved quantities serve as input parameters. In particular, we introduce the notion of infinitesimal concentration robustness, where the output variable remains nearly constant despite fluctuations in the conserved quantities. We provide several examples of chemical networks that illustrate our results both in deterministic and stochastic settings.

## Introduction

The original concept of homeostasis refers to a regulatory mechanism that keeps some variable close to a fixed value despite varying external factors (Bernard 1898). One classic example is the regulation of body temperature in mammals, regardless of environmental temperature changes. In Cannon (1926), this idea was further developed, and the term ‘homeostasis’ was coined. In modern science, the concept of homeostasis holds significant importance and is extensively studied in various fields, including molecular and population biology, biochemistry, and control theory.

Since various biological systems are often described by differential equations, one mathematical approach to describing homeostasis is to analyze a family of stable equilibria within a parameterized dynamical system of ordinary differential equations (ODEs). Homeostasis is observed when the stable equilibrium exhibits a relatively small change in response to a significantly larger change in an external parameter.

In Golubitsky and Stewart (2017), Golubitsky and Stewart used singularity theory to analyze homeostasis. They considered homeostasis in an input-output function associated with an ODE system with an input parameter ${\mathcal {I}}$ and an output variable $x_o$. Assuming that the input-output function $x_o ({\mathcal {I}})$ is well-defined in some neighborhood of a specific value ${\mathcal {I}}_0$, they introduced the concept of infinitesimal homeostasis, defined by the relation $\frac{d}{d {\mathcal {I}}} x_o ({\mathcal {I}}_0) = 0$. As the function value changes slowly near a critical point, this condition aligns with the intuitive notion of homeostasis.

This condition also has a natural interpretation in terms of sensitivity and identifiability: the vanishing derivative indicates that the output is locally insensitive to the input parameter, implying potential identifiability issues or robustness in parameter estimation (Minas and Rand 2019).

In Wang et al. (2021), Wang et al. studied infinitesimal homeostasis in input-output networks $\mathcal {G}$, where the input node $\iota$ is affected by the input parameter ${\mathcal {I}}$, and the output node is *o*. They considered an admissible family of ODEs associated with the input-output network $\mathcal {G}$. If we suppose that it admits a linearly stable family of equilibria, then the input-output function $x_o({\mathcal {I}})$ is well-defined for $\mathcal {G}$. They further demonstrated that the derivative of the input-output function, $\frac{d}{d {\mathcal {I}}} x_o ({\mathcal {I}}_0)$, depends on the determinant of the homeostasis matrix *H* (see (5) below for the definition). Therefore, the infinitesimal homeostasis can be determined when $\det (H) = 0$. In a recent paper (Duncan et al. 2023), Duncan et al. considered the homeostasis pattern in an input-output network $\mathcal {G}$. A homeostasis pattern is defined in Duncan et al. (2023) as a set of nodes in $\mathcal {G}$, including the output node *o*, with all nodes exhibiting infinitesimal homeostasis at a specific value ${\mathcal {I}}_0$.

In this article, we will be interested in special types of ODE systems that represent the dynamics of chemical reaction networks. Founded in the 1960s, chemical reaction network theory is an area of applied mathematics used to model the behavior of chemical and biochemical systems (Feinberg 1979, 2019). Due to its applications in modern biology, it has attracted the interest of both applied and pure mathematics communities.

In one of the first papers in the subject, Craciun and Deshpande analyzed homeostasis from the perspective of reaction networks (Craciun and Deshpande 2022). Given a reaction network *G*, they constructed a modified network $G'$ to determine whether *G* has the capacity for homeostasis and injectivity. Recently, in Yu and Sontag (2024) Yu and Sontag studied infinitesimal homeostasis, also known as quasi-adaptation, from a control theory perspective. They provided necessary and sufficient conditions when minors of a symbolic matrix had mixed signs, thereby establishing necessary conditions for quasi-adaptation.

Here, we will study a broader setting of infinitesimal homeostasis of reaction networks. Although some related work has already been done in Craciun and Deshpande (2022), the authors there considered only a relatively simple case where the stoichiometric subspace (see Definition 2.4) of the reaction network encompassed the entire Euclidean space of the appropriate dimension. In our analysis, we also include a more natural scenario where a reaction network admits conservation laws (see Definition 2.10).

Consider for example the reaction network consisting of a reversible pair as follows:Under the mass-action kinetics, the concentrations of species $x_1 (t), x_2 (t)$ satisfy

$$ \frac{d}{d t} (x_1 + x_2) = 0. $$

Therefore, there exists a constant *C* defined by the initial condition, such that

$$ x_1 (t) + x_2 (t) \equiv C. $$

First, we assume the constant *C* is fixed, the input parameter is a reaction rate constant ($k_1$ or $k_2$), and the output variable is the corresponding steady state of the species $X_2$. In Theorem 4.7 below, we provide a general way of verifying when infinitesimal homeostasis can happen in such a case. Next, we extend our input-output framework and replace the input parameter with the conservation constant *C* while keeping the same output variable. In this setting, we refer to such infinitesimal homeostasis as *infinitesimal concentration robustness* (see Definition 4.11). In Theorem 4.13 below, we provide a method to determine when infinitesimal concentration robustness occurs.

This method is based on the notions of the *modified Jacobian matrix* and the *modified homeostasis matrix*. The *modified Jacobian matrix* was introduced in Feliu and Wiuf (2012), where it is referred to as the Jacobian matrix of the “extended rate function.” It is used to formulate a Jacobian criterion that precludes the existence of multiple positive steady states. In particular, they show that a network is injective if and only if the determinant of this Jacobian does not vanish. Both matrices incorporate information from the original Jacobian matrix and the conservation laws. Using tools from combinatorial matrix theory, we establish a necessary and sufficient condition under which a mass-action system exhibits infinitesimal concentration robustness at a *non-degenerate equilibrium relative to its stoichiometric compatibility class*, as stated in Theorem 4.13.

We remark that the notion of such an equilibrium was introduced in Craciun and Feinberg (2010), where it was employed to study the existence of multiple equilibria in reaction networks. This concept was further explored in Banaji and Pantea (2016) in the context of injectivity and multistationarity in chemical reaction networks.

Finally, we consider infinitesimal homeostasis in the stochastic reaction networks, where the concentrations of species are random variables. Suppose a reaction network admits a stationary distribution, then we let the expectation of such a stationary distribution on a chosen random variable as the output. Inspired by the deterministic case, we provide a way to find the infinitesimal concentration robustness when the reaction network is first-order (See Definition 4.24).

### Paper outline

Section 2 introduces the terminology of mass-action systems, input-output networks, and infinitesimal homeostasis. In Section 3.1 we consider homeostasis in mass-action systems without the conservation laws. In Section 3.2 we discuss when infinitesimal homeostasis can happen in a complex-balanced system. Section 4.1 introduces the notion of a modified Jacobian matrix. In Section 4.2 we consider reaction networks under the conservation laws, and state two main theorems of this paper, Theorems 4.7 and 4.13, which provide a way to compute infinitesimal homeostasis and infinitesimal concentration robustness, respectively. In Section 4.3 we discuss infinitesimal homeostasis in the stochastic reaction networks. Section 5 contains a brief discussion of some possible generalizations on different types of infinitesimal homeostasis and general stochastic reaction networks.

### Notation

Throughout this work, we let $\mathbb {R}_{\ge 0}^n$ and $\mathbb {R}_{> 0}^n$ denote *n*-dimensional Euclidean non-negative and positive orthants, respectively. Similarly, $\mathbb {Z}_{\ge 0}^n$ represents the set of vectors with non-negative integer components. We let $\textbf{e}_i = (0, \ldots , 0, 1, 0, \ldots , 0)$ represent the standard basis vector where the 1 appears in the *i*-th position. Vectors are typically denoted by $\boldsymbol{x}$ or $\boldsymbol{y}$.

## Background

In this section, we will begin by introducing key terminology and results in the reaction network theory. We will then review concepts and findings related to homeostasis in the input-output network.

### Euclidean embedded graphs and mass-action systems

In this subsection, we introduce a directed graph in $\mathbb {R}^n$ known as the *Euclidean embedded graph* and illustrate how to define the associated *mass-action system* based on this graph structure.

#### Definition 2.1

( Craciun (2015); Craciun and Deshpande (2020))

- A **reaction network** $G = (V, E)$, also known as a **Euclidean embedded graph (or E-graph)**, is a directed graph in $\mathbb {R}^n$, where $V \subset \mathbb {R}^n$ denotes a finite set of **vertices**, and $E \subseteq V \times V$ denotes a finite set of **edges**. In this context, we assume that there are no isolated vertices, no self-loops, and at most one edge between any pair of ordered vertices.
- Let $V = \{ \boldsymbol{y}_1, \ldots , \boldsymbol{y}_m \}$. A directed edge $(\boldsymbol{y}_i, \boldsymbol{y}_j) \in E$, referred to as a **reaction** in the network, is also denoted by $\boldsymbol{y}_i \rightarrow \boldsymbol{y}_j$, where $\boldsymbol{y}_i$ and $\boldsymbol{y}_j$ are termed the **source vertex** and **target vertex**, respectively. Furthermore, we define the **reaction vector** associated with the reaction $\boldsymbol{y}_i \rightarrow \boldsymbol{y}_j$ to be $\boldsymbol{y}_j - \boldsymbol{y}_i \in \mathbb {R}^n$.

#### Definition 2.2

Let $G=(V, E)$ be an E-graph.

- The set of vertices *V* is partitioned into its connected components, which are also referred to as **linkage classes**.
- A connected component $L \subseteq V$ is termed **strongly connected** if every edge in *L* is part of a directed cycle within *L*. Moreover, $G = (V, E)$ is termed **weakly reversible** if each of its connected components is strongly connected.

#### Example 2.3

**Fig. 1:**
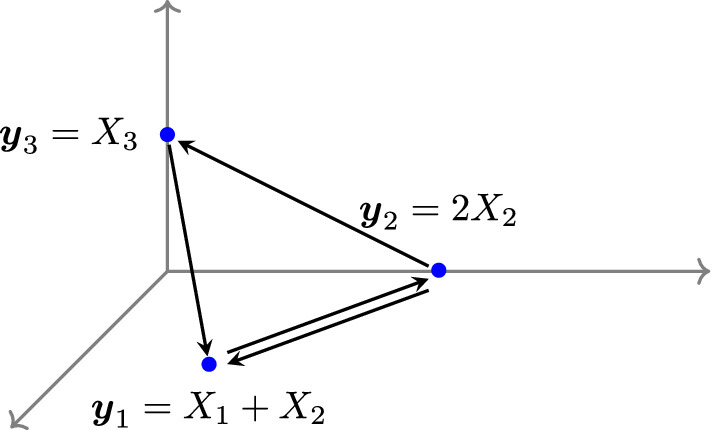
This E-graph $G = (V, E)$ is weakly reversible and consists of a single linkage class

Figure 1 depicts an example of a reaction network represented as an E-graph $G = (V, E)$. There are three vertices:

$$\begin{aligned} V = \{ \boldsymbol{y}_1, \ \boldsymbol{y}_2, \ \boldsymbol{y}_3 \}, \end{aligned}$$

and four directed edges between vertices:

$$\begin{aligned} E = \{ \boldsymbol{y}_1 \rightarrow \boldsymbol{y}_2, \ \boldsymbol{y}_2 \rightarrow \boldsymbol{y}_1, \ \boldsymbol{y}_2 \rightarrow \boldsymbol{y}_3, \ \boldsymbol{y}_3 \rightarrow \boldsymbol{y}_1 \}. \end{aligned}$$

$$\square $$

#### Definition 2.4

( Feinberg (1979)) Let $G=(V, E)$ be an E-graph. Define a **reaction rate vector** $\boldsymbol{k}$ as

$$ \boldsymbol{k}= (k_{\boldsymbol{y}_i \rightarrow \boldsymbol{y}_j})_{(i,j) \in E} \in \mathbb {R}^{|E|}_{>0}, $$

where $k_{\boldsymbol{y}_i \rightarrow \boldsymbol{y}_j}$ or $k_{ij}$ is the **reaction rate constant** associated with the edge $(i,j): \boldsymbol{y}_i \rightarrow \boldsymbol{y}_j \in E$. The **associated mass-action dynamical system** generated by $(G, \boldsymbol{k})$ is a dynamical system on $\mathbb {R}_{>0}^n$ defined by

1 $$\begin{aligned} \frac{d \boldsymbol{x}}{d t} = \sum _{\boldsymbol{y}_i \rightarrow \boldsymbol{y}_j \in E}k_{\boldsymbol{y}_i \rightarrow \boldsymbol{y}_j} \boldsymbol{x}^{\boldsymbol{y}_i}(\boldsymbol{y}_j - \boldsymbol{y}_i), \end{aligned}$$

where $\boldsymbol{x}^{\boldsymbol{y}}:= x_1^{y_{1}} x_2^{y_{2}} \ldots x_n^{y_{n}}$.^1^ Furthermore, we define the **stoichiometric subspace** of *G* as the span of the reaction vectors of *G*, represented by

$$\begin{aligned} \mathcal {S}= {{\,\textrm{span}\,}}\{ \boldsymbol{y}_j - \boldsymbol{y}_i: \boldsymbol{y}_i \rightarrow \boldsymbol{y}_j \in E \}. \end{aligned}$$

In Sontag (2001), it is shown that the positive orthant $\mathbb {R}_{>0}^n$ is forward-invariant when $V \subset \mathbb {Z}_{\ge 0}^n$. Therefore, in this context we always assume $V \subset \mathbb {Z}_{\ge 0}^n$, ensuring that any solution to (1) with initial condition $\boldsymbol{x}_0 \in \mathbb {R}_{>0}^n$ and $V \subset \mathbb {Z}_{\ge 0}^n$ is confined to the set

$$ \mathcal {S}_{\boldsymbol{x}_0} := (\boldsymbol{x}_0 + \mathcal {S}) \cap \mathbb {R}_{>0}^n, $$

which defines the **stoichiometric compatibility class** of *G* at $\boldsymbol{x}_0$.

#### Example 2.5

**Fig. 2:**
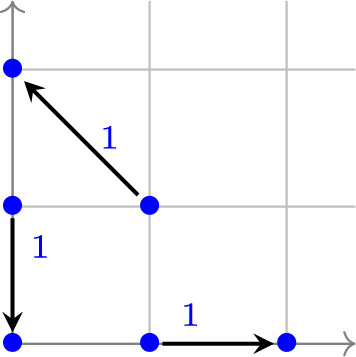
The E-graph *G* consists of 6 vertices and 3 edges. Under mass-action kinetics, this network corresponds to the classical Lotka–Volterra model for population dynamics

The E-graph $G = (V, E)$ in Figure 2 has 3 edges and 6 vertices. Given the reaction rate constants shown in Figure 2, the associated mass-action dynamical system $(G, \boldsymbol{k})$ is

$$\begin{aligned} \begin{aligned} \frac{d \boldsymbol{x}}{d t}&= x_1 \begin{pmatrix} 1 \\ 0 \end{pmatrix} + x_1 x_2 \begin{pmatrix} -1 \\ 1 \end{pmatrix} + x_2 \begin{pmatrix} 0 \\ -1 \end{pmatrix} = \begin{pmatrix} x_1 - x_1 x_2 \\ x_1 x_2 - x_2 \end{pmatrix}. \end{aligned} \end{aligned}$$

This is the Lotka–Volterra population dynamics model (Freedman 1982).

$$\square $$

### Complex-balanced systems

In general, mass-action systems exhibit diverse dynamics. In this section, we introduce a special class of systems: *complex-balanced dynamical systems*. The significance of complex-balanced dynamical systems derives from the strong stability properties of their positive equilibria, known as the complex-balanced equilibria.

#### Definition 2.6

Let $(G, \boldsymbol{k})$ be a mass-action system as follows:

$$\begin{aligned} \frac{d \boldsymbol{x}}{d t} = \sum _{\boldsymbol{y}\rightarrow \boldsymbol{y}' \in E}k_{\boldsymbol{y}\rightarrow \boldsymbol{y}'} \boldsymbol{x}^{\boldsymbol{y}}(\boldsymbol{y}' - \boldsymbol{y}). \end{aligned}$$

A point $\boldsymbol{x}^* \in \mathbb {R}_{>0}^n$ is termed a **positive equilibrium** of $(G, \boldsymbol{k})$ if it satisfies

$$ \sum _{\boldsymbol{y}\rightarrow \boldsymbol{y}' \in E}k_{\boldsymbol{y}\rightarrow \boldsymbol{y}'} (\boldsymbol{x}^*)^{\boldsymbol{y}}(\boldsymbol{y}' - \boldsymbol{y}) = \textbf{0}. $$

A positive equilibrium $\boldsymbol{x}^* \in \mathbb {R}_{>0}^n$ is called a **complex-balanced equilibrium** of $(G, \boldsymbol{k})$ if for every vertex $\boldsymbol{y}\in V$,

$$\begin{aligned} \sum _{\boldsymbol{y}\rightarrow \hat{\boldsymbol{y}} \in E} k_{\boldsymbol{y}\rightarrow \hat{\boldsymbol{y}}} (\boldsymbol{x}^*)^{\boldsymbol{y}} = \sum _{\boldsymbol{y}' \rightarrow \boldsymbol{y}\in E}k_{\boldsymbol{y}' \rightarrow \boldsymbol{y}} (\boldsymbol{x}^*)^{\boldsymbol{y}'}. \end{aligned}$$

We say that the pair $(G, \boldsymbol{k})$ **satisfies the complex-balanced conditions** if it has a complex-balanced equilibrium. In this case, the mass-action system generated by $(G, \boldsymbol{k})$ is referred to as a **complex-balanced system**.

Complex-balanced systems exhibit both algebraic and dynamical properties. The following classical theorem highlights key properties of complex-balanced systems.

#### Theorem 2.7

( Horn and Jackson (1972)) Let $(G, \boldsymbol{k})$ be a complex-balanced system with a positive equilibrium $\boldsymbol{x}^* \in \mathbb {R}_{>0}^n$. Then we have the following:

- All positive equilibria of complex-balanced systems are complex-balanced equilibria, and there is exactly one equilibrium within each stoichiometric compatibility class of the system.
- Every complex-balanced equilibrium $\boldsymbol{x}$ satisfies
  $$ \ln \boldsymbol{x}- \ln \boldsymbol{x}^* \in \mathcal {S}^\perp , $$
  where $\mathcal {S}$ is the stoichiometric subspace of *G*, and $\ln (\cdot )$ is defined component-wise on vectors and element-wise on sets of vectors.
- Every complex-balanced equilibrium is asymptotically stable within its stoichiometric compatibility class.

$$\square $$

The necessary and sufficient conditions for the system $(G, \boldsymbol{k})$ to be complex-balanced are: (1) the graph $G = (V, E)$ is weakly reversible, and (2) the reaction rate vector $\boldsymbol{k}$ satisfies a specific set of algebraic equations, the number of which is determined by a non-negative integer known as the *deficiency*.

#### Definition 2.8

Let $G = (V, E)$ be an E-graph with $\ell$ linkage classes, and let *S* be its associated stoichiometric subspace. The **deficiency** of $G = (V, E)$ is an integer defined as

$$ \delta = |V| - \ell - \dim \mathcal {S}. $$

Under mass-action kinetics, networks with low deficiency exhibit special dynamical properties. Specifically, the Deficiency Zero Theorem states that weakly reversible networks with a deficiency of zero are complex-balanced for any choice of rate constants (Horn 1972).

#### Theorem 2.9

(Deficiency Zero Theorem, Horn (1972))

Let $G = (V, E)$ be an E-graph. The mass-action system $(G, \boldsymbol{k})$ is complex-balanced for any choice of reaction rate vector $\boldsymbol{k}\in \mathbb {R}^{|E|}_{>0}$ if and only if the E-graph *G* is weakly reversible and has zero deficiency. $\square$

Consider Example 2.3, the E-graph *G* from Figure 1 is weakly reversible and consists of three vertices $(|V| = 3)$ in one linkage class $(\ell =1)$. Additionally, the dimension of the stoichiometric subspace is two $(\dim \mathcal {S}= 2)$. Thus, the deficiency is $\delta = 3 - 1 - 2 = 0$. From Theorem 2.9, the mass-action system $(G, \boldsymbol{k})$ is always complex-balanced for any choice of reaction rate vector $\boldsymbol{k}\in \mathbb {R}^{|E|}_{>0}$.

#### Definition 2.10

Let $G = (V, E)$ be an E-graph. A function $\phi (\boldsymbol{x})$ is termed a **conservation law** if it is a first integral of the system (1) for every reaction rate vector $\boldsymbol{k}$, that is,

$$ \sum \limits ^{n}_{i=1} \frac{\partial \phi }{\partial x_i} (\boldsymbol{x}) \frac{d x_i}{d t} = 0. $$

Consider the mass-action system $(G, \boldsymbol{k})$ given by (1), and let $\mathcal {S}$ be the associated stoichiometric subspace. For any vector $\boldsymbol{u}= (u_1, \ldots , u_n)^{\intercal } \in \mathcal {S}^\perp$, we have

2 $$\begin{aligned} \sum \limits ^{n}_{i=1} u_i \frac{d x_i}{d t} = 0, \end{aligned}$$

and thus $\sum \limits ^{n}_{i=1} u_i x_i$ is a linear conservation law. For simplicity, we let $\boldsymbol{u}= (u_1, \ldots , u_n)^{\intercal }$ denote the linear conservation law $\sum \limits ^{n}_{i=1} u_i x_i$.

#### Example 2.11

Revisiting Example 2.3, the stoichiometric subspace $\mathcal {S}$ of the E-graph *G* from Figure 1 is given by

$$ \mathcal {S}= {{\,\textrm{span}\,}}\{ \boldsymbol{y}_2 - \boldsymbol{y}_1, \boldsymbol{y}_1 - \boldsymbol{y}_2, \boldsymbol{y}_1 - \boldsymbol{y}_3, \boldsymbol{y}_3 - \boldsymbol{y}_2 \} = {{\,\textrm{span}\,}}\{ (1, -1, 0)^{\intercal }, (0,-1,1)^{\intercal } \}. $$

Then we obtain that $(1, 1, 1)^{\intercal } \in \mathcal {S}^\perp$ and derive a conservation law

$$ \phi (x) = x_1 + x_2 + x_3. $$

$$\square $$

### Infinitesimal homeostasis

In this section, we continue to introduce some key concepts, including *input-output systems*, *input-output functions*, and *infinitesimal homeostasis*. Some of our discussion is based on Golubitsky and Stewart (2017); Wang et al. (2021).

#### Definition 2.12

Let $\boldsymbol{x}= (x_1, x_2, \ldots , x_n) \in \mathbb {R}^n$ be the vector of state variables, and let ${\mathcal {I}}\in \mathbb {R}$ be the **input parameter**. The **input-output system** is a dynamical system on $\mathbb {R}_{>0}^n$ defined by

3 $$\begin{aligned} \begin{aligned} \frac{d x_1}{d t}&= f_1 (x_1, \ldots , x_n, {\mathcal {I}}), \\ \frac{d x_2}{d t}&= f_2 (x_1, \ldots , x_n), \\ &\ \vdots \\ \frac{d x_n}{d t}&= f_n (x_1, \ldots , x_n), \end{aligned} \end{aligned}$$

where $\boldsymbol{f}(\boldsymbol{x}, {\mathcal {I}}) = (f_1 (\boldsymbol{x}, {\mathcal {I}}), \ldots , f_n (\boldsymbol{x}))$ is a smooth family of mappings on the state space $\mathbb {R}^n$. For notational convenience, we assume both above and throughout the paper that the input parameter ${\mathcal {I}}$ appears only in the first equation of (3), and that the state variable $x_n$ serves as the **output parameter**.

#### Remark 2.13

Throughout the paper, we assume that the function $f_n (\boldsymbol{x})$ of (3) is not identically zero. Otherwise, $\frac{d x_n}{d t} \equiv 0$ implies that the output parameter $x_n$ is fixed within the stoichiometric compatibility class of the trajectory, which represents a trivial case.

The input-output system (3) can be written as

4 $$\begin{aligned} \frac{d \boldsymbol{x}}{d t} = \boldsymbol{f}(\boldsymbol{x}, {\mathcal {I}}). \end{aligned}$$

In Wang et al. (2021), it is shown that when for some input ${\mathcal {I}}_0$ the system $\frac{d \boldsymbol{x}}{d t} = \boldsymbol{f}(\boldsymbol{x}, {\mathcal {I}}_0)$ has a *hyperbolic* equilibrium (or *non-degenerate* equilibrium^2^) $\boldsymbol{x}= \boldsymbol{x}_0$, then the implicit function theorem implies the existence of a unique family of equilibria

$$ \boldsymbol{x}({\mathcal {I}}) = (x_1 ({\mathcal {I}}), \ldots , x_n ({\mathcal {I}})), $$

such that $\boldsymbol{x}({\mathcal {I}}_0) = \boldsymbol{x}_0$ and $\boldsymbol{f}(\boldsymbol{x}({\mathcal {I}}), {\mathcal {I}}) = 0$ for every ${\mathcal {I}}$ near ${\mathcal {I}}_0$. With the function $x_n = x_n({\mathcal {I}})$, we can illustrate the relationship between the output parameter $x_n$ and the input parameter ${\mathcal {I}}$.

*Homeostasis* occurs in a system of differential equations when the output parameter $x_n$ remains approximately constant while the input parameter ${\mathcal {I}}$ varies. In Golubitsky and Stewart (2017), the concept of *infinitesimal homeostasis* is introduced as follows.

#### Definition 2.14

(Golubitsky and Stewart (2017))

Given the input-output system (3), suppose the mapping ${\mathcal {I}}\mapsto x_n ({\mathcal {I}})$ is well-defined in a neighborhood of ${\mathcal {I}}_0$, then $x_n ({\mathcal {I}})$ is called the **input-output function**. Moreover, **infinitesimal homeostasis** occurs at ${\mathcal {I}}_0$ if

$$ \frac{d}{d {\mathcal {I}}} x_n ({\mathcal {I}}_0) = 0. $$

Since a function varies slowly near a stationary point, infinitesimal homeostasis is a *sufficient* condition for homeostasis over some interval of the input parameter (Golubitsky and Stewart 2017).

In Wang et al. (2021), the authors proved that infinitesimal homeostasis occurs when the determinant of the homeostasis matrix *H* is 0. In this context, the **homeostasis matrix** *H* is obtained from the Jacobian matrix *J* of (3) by deleting its row corresponding to $x_1$ and its column corresponding to $x_n$. More precisely,

5 $$\begin{aligned} J = \begin{pmatrix} f_{1, 1} & \ldots & f_{1, n-1} & f_{1, n} \\ f_{2, 1} & \ldots & f_{2, n-1} & f_{2, n} \\ \vdots \ \ \  & & & \vdots \ \ \ \\ f_{n, 1} & \ldots & f_{n, n-1} & f_{n, n} \end{pmatrix} \qquad \Longrightarrow \qquad H = \begin{pmatrix} f_{2, 1} & \ldots & f_{2, n-1} \\ \vdots \ \ \  & & \vdots \ \ \ \ \\ f_{n, 1} & \ldots & f_{n, n-1} \end{pmatrix}, \end{aligned}$$

where $f_{j,\ell }$ denotes the partial derivative of the function $f_j$ with respect to the state variable $x_\ell$.

An intuition behind the introduction of the matrix *H* is as follows: The family of equilibria satisfies

$$ \textbf{f}({\mathcal {I}}, \boldsymbol{x}({\mathcal {I}})) = \textbf{0} \ \text { for every } {\mathcal {I}}\text { near } {\mathcal {I}}_0. $$

Differentiating both sides with respect to ${\mathcal {I}}$ and noting that the input parameter ${\mathcal {I}}$ appears only in $f_1$, we obtain

$$ J \frac{d}{d {\mathcal {I}}} \boldsymbol{x}({\mathcal {I}}) = (\frac{\partial {f_1}}{\partial {\mathcal {I}}}, 0, \ldots , 0)^{\intercal } \ \text { for every } {\mathcal {I}}\text { near } {\mathcal {I}}_0. $$

The *H* matrix is then simply a result of the expansion of the determinant of *J* via Cramer’s rule. A similar approach is employed in the proofs of Theorems 4.7 and 4.13.

#### Theorem 2.15

(Frobenius–König theory (Brualdi and Ryser 1991))

Let *H* be the homeostasis matrix defined in (5) of an input-output network. There are permutation matrices *P* and *Q* such that *PHQ* is block upper triangular with irreducible square blocks $B_1, \ldots , B_m$, that is,

$$ PHQ = \begin{pmatrix} B_1 & \cdots & * & \cdots & * \\ & \ddots & & & \\ 0 & & B_{\eta } & \cdots & * \\ & & & \ddots & \\ 0 & & 0 & & B_{m} \end{pmatrix}. $$

Then

6 $$\begin{aligned} \det (H) = \det (B_1) \cdots \det (B_m). \end{aligned}$$

$$\square $$

From Theorem 2.15, computing the determinant of the homeostasis matrix reduces to computing the determinant of these irreducible blocks.

#### Definition 2.16

( Wang et al. (2021))

Each irreducible square block $B_{\eta }$ in (6) is referred to as a **homeostasis block**. Moreover, an infinitesimal homeostasis is said to be of **homeostasis type** $B_{\eta }$ if

$$\begin{aligned} \det (B_{\eta }) = 0 \ \text { and } \ \det (B_{\xi }) \ne 0 \ \text { for all } \ \xi \ne \eta . \end{aligned}$$

## Basic properties

In this section, we briefly review some basic properties of the mathematical theory of homeostasis as applied to (deterministic) mass-action systems and relevant to our results in the subsequent sections. We begin by considering the mass-action systems whose stoichiometric subspaces encompass the entire space $\mathbb {R}^n$.

### Homeostasis in mass-action systems with $\mathcal {S}= \mathbb {R}^n$

To examine homeostasis in mass-action systems, we need to construct an input-output system based on Definition 2.12 as follows:

7 $$\begin{aligned} \begin{aligned} \frac{d x_1}{d t}&= f_1 (x_1, \ldots , x_n, {\mathcal {I}}), \\ \frac{d x_2}{d t}&= f_2 (x_1, \ldots , x_n), \\ &\ \vdots \\ \frac{d x_n}{d t}&= f_n (x_1, \ldots , x_n), \end{aligned} \end{aligned}$$

where $f_1, \ldots , f_n$ are smooth mappings satisfying the mass-action kinetics in Definition 2.4.

Recall that, for consistency of notation, we assume the input parameter ${\mathcal {I}}$ appears only in the first equation of (7). As a starting point, we introduce the following simple input model, which will be used throughout this section. According to the mass-action kinetics in Definition 2.4, we consider an inflow reaction in which the reaction rate constant serves as the input parameter, given by

8 $$\begin{aligned} \emptyset \xrightarrow {{\mathcal {I}}} a X_1, \end{aligned}$$

where $a \in \mathbb {Z}_{>0}$, and $x_n$ is the output parameter.

Suppose that the system (7) has a non-degenerate equilibrium $\boldsymbol{x}= \boldsymbol{x}_0$ when ${\mathcal {I}}= {\mathcal {I}}_0$, and the input-output function $x_n ({\mathcal {I}})$ is well-defined in a neighborhood of ${\mathcal {I}}_0$. From Definition 2.14, along with Wang et al. (2021), infinitesimal homeostasis occurs at ${\mathcal {I}}_0$ when the determinant of the homeostasis matrix *H* defined in (5) is 0.

#### Example 3.1

**Fig. 3:**
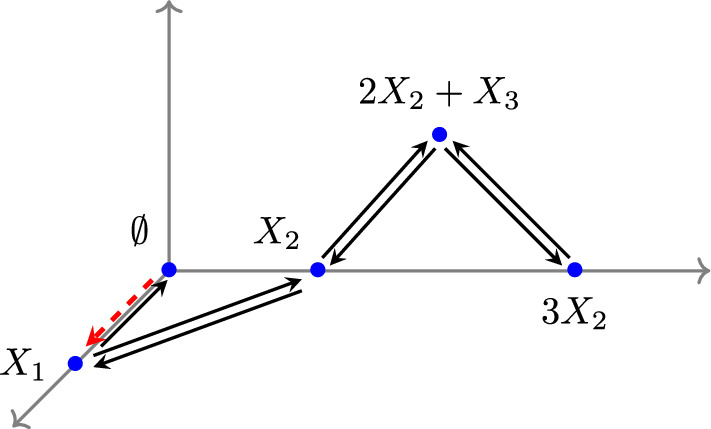
The E-graph $G = (V, E)$ contains an inflow reaction with the input parameter (reaction rate constant) ${\mathcal {I}}$ depicted by the red dashed arrow

Consider the E-graph $G = (V, E)$ in Figure 3. Suppose all reaction rate constants are equal to 1 except for the rate of the inflow reaction, which is ${\mathcal {I}}$, represented as

$$ \emptyset \xrightarrow {{\mathcal {I}}} X_1. $$

The mass-action system is then given by

9 $$\begin{aligned} \begin{aligned}&\frac{d x_1}{dt} = - 2 x_1 + x_2 + {\mathcal {I}}, \\ &\frac{d x_2}{dt} = x_1 - (x_2)^3, \\ &\frac{d x_3}{dt} = x_2 + (x_2)^3 - 2 (x_2)^2 x_3. \end{aligned} \end{aligned}$$

Let $\boldsymbol{x}^* = (x^*_1, x^*_2, x^*_3)$ be the equilibrium with corresponding ${\mathcal {I}}= {\mathcal {I}}_0$, then we have

$$ 2 x^*_1 = x^*_2 + {\mathcal {I}}_0, \ \ x^*_1 = (x^*_2)^3, \ \ x^*_2 + (x^*_2)^3 = 2 (x^*_2)^2 x^*_3. $$

Moreover, we obtain the homeostasis matrix as follows:

$$\begin{aligned} H = \begin{pmatrix} 1 & - 3 (x_2)^2 \\ 0 & 1 + 3 (x_2)^2 - 4 x_2 x_3 \end{pmatrix}, \end{aligned}$$

and thus infinitesimal homeostasis occurs at $\boldsymbol{x}^* = (x^*_1, x^*_2, x^*_3)$ when

$$ \det (H) = 1 + 3 (x^*_2)^2 - 4 x^*_2 x^*_3 = 0. $$

We consider the equilibrium $(x^*_1, x^*_2, x^*_3) = (1, 1, 1)$ which satisfies the above equations with corresponding ${\mathcal {I}}_0 = 1$. We further verify that the Jacobian matrix is non-singular at this point, indicating it is a non-degenerate equilibrium. Therefore, we conclude that infinitesimal homeostasis occurs at ${\mathcal {I}}_0 = 1$.

$$\square $$

### Infinitesimal homeostasis in complex-balanced systems

In Sections 2.3 and 3.1, both the input-output function $x_n'({\mathcal {I}}_0)$ and infinitesimal homeostasis are well-defined around a non-degenerate equilibrium when ${\mathcal {I}}= {\mathcal {I}}_0$. On the other hand, given any complex-balanced system $(G, \boldsymbol{k})$ with $\mathcal {S}= \mathbb {R}^n$, Theorem 2.7 shows that every complex-balanced equilibrium is asymptotically stable and non-degenerate.

The above fact brings up the following question: *Given a complex-balanced system* $(G, \boldsymbol{k})$ *with an input parameter* ${\mathcal {I}}$, *when does infinitesimal homeostasis occurs for some* ${\mathcal {I}}= {\mathcal {I}}_0$?

We begin by formulating an input-output system (7), where we impose certain constraints on $(G, \boldsymbol{k})$ and the input parameter ${\mathcal {I}}$ as follows:

- *G* is weakly reversible and the associated stoichiometric subspace $\mathcal {S}= \mathbb {R}^n$.
- *G* contains only one inflow reaction with the reaction rate constant as the input parameter, given by
  10 $$\begin{aligned} \emptyset \xrightarrow {{\mathcal {I}}} a X_1 \ \text { with } \ a \in \mathbb {Z}_{>0}. \end{aligned}$$
  Hence, for any other reaction $\boldsymbol{y}\rightarrow \boldsymbol{y}' \in E$, the source vertex $\boldsymbol{y}\ne \emptyset$.
- For every reaction $\boldsymbol{y}\rightarrow \boldsymbol{y}' \in E$ except the inflow reaction (10), it has a fixed reaction rate constant $k_{\boldsymbol{y}\rightarrow \boldsymbol{y}'}$.

For simplicity, we denote this input-output system by $(G, \boldsymbol{k}, {\mathcal {I}})$. To address the above question regarding $(G, \boldsymbol{k}, {\mathcal {I}})$, we construct an associated mass-action system $(\hat{G}, \hat{\boldsymbol{k}})$ as follows:

**Step 1. ** Let $\hat{V} = V \ \backslash \ \{ \emptyset \}$. For every reaction $\boldsymbol{y}\xrightarrow {k} \boldsymbol{y}' \in E$ where $\boldsymbol{y}\ne \emptyset , \boldsymbol{y}' \ne \emptyset$, we add

$$ \boldsymbol{y}\xrightarrow {k} \boldsymbol{y}' \in \hat{E}. $$

We omit the reaction $\emptyset \rightarrow a X_1$, and also, if it exists, the reaction $a X_1 \rightarrow \emptyset$.

**Step 2. ** For every reaction $\boldsymbol{y}\xrightarrow {k} \emptyset \in E$ with $\boldsymbol{y}\ne a X_1$, we replace this reaction in $\hat{G}$ with

$$ \boldsymbol{y}\xrightarrow {k} a X_1 \in \hat{E}. $$

If both reactions $y \xrightarrow {k_1} \emptyset \in E$ and $y \xrightarrow {k_2} a X_1 \in E$ with $\boldsymbol{y}\ne a X_1$, then we add the following reaction to $\hat{E}$:

$$ y \xrightarrow {k_1 + k_2} a X_1 \in \hat{E}. $$

$$\square $$

#### Remark 3.2

The mathematical intuition behind the construction of the associated mass-action system $(\hat{G}, \hat{\boldsymbol{k}})$ from an input-output system $(G, \boldsymbol{k}, {\mathcal {I}})$ is that both systems share the same homeostasis matrix (see Proposition 3.3). The logic behind this construction parallels the approach in Craciun and Deshpande (2022), where a modified network was introduced to determine whether the original network admits homeostasis.

Moreover, we will show that if $(G, \boldsymbol{k}, {\mathcal {I}})$ is complex-balanced for some input ${\mathcal {I}}= {\mathcal {I}}_0$, then the associated system $(\hat{G}, \hat{\boldsymbol{k}})$ is also complex-balanced. This enables the analysis of the homeostatic and complex-balanced behavior of $(G, \boldsymbol{k}, {\mathcal {I}})$ via $(\hat{G}, \hat{\boldsymbol{k}})$.

Now we demonstrate that it suffices to examine the mass-action system $(\hat{G}, \hat{\boldsymbol{k}})$ to determine whether the system $(G, \boldsymbol{k}, {\mathcal {I}})$ exhibits infinitesimal homeostasis at ${\mathcal {I}}_0$, with $(G, \boldsymbol{k}, {\mathcal {I}}_0)$ being complex-balanced.

#### Proposition 3.3

Assume that $(\hat{G}, \hat{\boldsymbol{k}})$ is a complex-balanced system with stoichiometric subspace $\mathcal {S}= \mathbb {R}^n$, and assume it admits a positive equilibrium $\boldsymbol{x}^* \in \mathbb {R}_{>0}^n$. Suppose that the associated homeostasis matrix $\hat{H}$ satisfies $\det (\hat{H}) = 0$ when $\boldsymbol{x}= \boldsymbol{x}^*$. There exists ${\mathcal {I}}_0 > 0$ such that the input-output system $(G, \boldsymbol{k}, {\mathcal {I}})$ exhibits infinitesimal homeostasis at ${\mathcal {I}}_0$ and $(G, \boldsymbol{k}, {\mathcal {I}}_0)$ is a complex-balanced system.

#### Proof

For simplicity, we denote the inflow reaction in *G* by

$$ \emptyset \xrightarrow {{\mathcal {I}}} \boldsymbol{y}_0 := a X_1. $$

First, we show $(G, \boldsymbol{k}, {\mathcal {I}}_0)$ is a complex-balanced system. From the assumption, $(\hat{G}, \hat{\boldsymbol{k}})$ has a complex-balanced equilibrium $\boldsymbol{x}^* \in \mathbb {R}_{>0}^n$, that is,

11 $$\begin{aligned} \sum _{\boldsymbol{y}_0 \rightarrow \boldsymbol{y}\in \hat{E}} \hat{k}_{\boldsymbol{y}_0 \rightarrow \boldsymbol{y}} (\boldsymbol{x}^*)^{\boldsymbol{y}_0} = \sum _{\boldsymbol{y}' \rightarrow \boldsymbol{y}_0 \in \hat{E}} \hat{k}_{\boldsymbol{y}' \rightarrow \boldsymbol{y}_0} (\boldsymbol{x}^*)^{\boldsymbol{y}'}. \end{aligned}$$

Since *G* is weakly reversible, there are some reactions

12 $$\begin{aligned} \boldsymbol{y}\xrightarrow {k} \emptyset \in E. \end{aligned}$$

Under this construction, for $\boldsymbol{y}\ne \boldsymbol{y}_0$, the reactions in (12) are replaced in $\hat{E}$ as follows:

$$ \boldsymbol{y}\xrightarrow {k} \boldsymbol{y}_0 \in \hat{E} \ \text { with } \ \boldsymbol{y}\ne \boldsymbol{y}_0, $$

and thus for every reaction $\boldsymbol{y}\rightarrow \boldsymbol{y}_0 \in \hat{E}$ with $\boldsymbol{y}\ne \boldsymbol{y}_0$, we have

13 $$\begin{aligned} \hat{k}_{\boldsymbol{y}\rightarrow \boldsymbol{y}_0} = k_{\boldsymbol{y}\rightarrow \boldsymbol{y}_0} + k_{\boldsymbol{y}\rightarrow \emptyset }. \end{aligned}$$

Let ${\mathcal {I}}_0 = \sum _{\boldsymbol{y}\rightarrow \emptyset \in E} k_{\boldsymbol{y}\rightarrow \emptyset } (\boldsymbol{x}^*)^{\boldsymbol{y}}$, we claim that the input-output system $(G, \boldsymbol{k}, {\mathcal {I}}_0)$ has a complex-balanced equilibrium $\boldsymbol{x}^*$.

For the vertex $\emptyset$, since there is only one inflow reaction $\emptyset \rightarrow \boldsymbol{y}_0 \in E$, then

$$ \sum \limits _{\emptyset \rightarrow \boldsymbol{y}\in E} k_{\emptyset \rightarrow \boldsymbol{y}} = {\mathcal {I}}_0 = \sum \limits _{\boldsymbol{y}\rightarrow \emptyset \in E} k_{\boldsymbol{y}\rightarrow \emptyset } (\boldsymbol{x}^*)^{\boldsymbol{y}}. $$

For the vertex $\boldsymbol{y}_0$, from the setting of ${\mathcal {I}}_0$ and (13) we have

$$\begin{aligned} \begin{aligned} \sum _{\boldsymbol{y}' \rightarrow \boldsymbol{y}_0 \in E} k_{\boldsymbol{y}' \rightarrow \boldsymbol{y}_0} (\boldsymbol{x}^*)^{\boldsymbol{y}'}&= {\mathcal {I}}_0 + \sum _{\boldsymbol{y}' \rightarrow \boldsymbol{y}_0 \in E, \ \boldsymbol{y}' \ne \emptyset } k_{\boldsymbol{y}' \rightarrow \boldsymbol{y}_0} (\boldsymbol{x}^*)^{\boldsymbol{y}'} \\ &= \sum \limits _{\boldsymbol{y}\rightarrow \emptyset \in E} k_{\boldsymbol{y}\rightarrow \emptyset } (\boldsymbol{x}^*)^{\boldsymbol{y}} + \sum _{\boldsymbol{y}' \rightarrow \boldsymbol{y}_0 \in E, \ \boldsymbol{y}' \ne \emptyset } k_{\boldsymbol{y}' \rightarrow \boldsymbol{y}_0} (\boldsymbol{x}^*)^{\boldsymbol{y}'} \\ &= k_{\boldsymbol{y}_0 \rightarrow \emptyset } (\boldsymbol{x}^*)^{\boldsymbol{y}_0} + \sum _{\boldsymbol{y}' \rightarrow \boldsymbol{y}_0 \in \hat{E}} \hat{k}_{\boldsymbol{y}' \rightarrow \boldsymbol{y}_0} (\boldsymbol{x}^*)^{\boldsymbol{y}'}. \end{aligned} \end{aligned}$$

Together with (11), we obtain that

$$\begin{aligned} \begin{aligned}&k_{\boldsymbol{y}_0 \rightarrow \emptyset } (\boldsymbol{x}^*)^{\boldsymbol{y}_0} + \sum _{\boldsymbol{y}' \rightarrow \boldsymbol{y}_0 \in \hat{E}} \hat{k}_{\boldsymbol{y}' \rightarrow \boldsymbol{y}_0} (\boldsymbol{x}^*)^{\boldsymbol{y}'} \\ &= k_{\boldsymbol{y}_0 \rightarrow \emptyset } (\boldsymbol{x}^*)^{\boldsymbol{y}_0} + \sum _{\boldsymbol{y}_0 \rightarrow \boldsymbol{y}\in \hat{E}} \hat{k}_{\boldsymbol{y}_0 \rightarrow \boldsymbol{y}} (\boldsymbol{x}^*)^{\boldsymbol{y}_0} = \sum _{\boldsymbol{y}_0 \rightarrow \boldsymbol{y}\in E} k_{\boldsymbol{y}_0 \rightarrow \boldsymbol{y}} (\boldsymbol{x}^*)^{\boldsymbol{y}_0}. \end{aligned} \end{aligned}$$

For the remaining vertices in *G*, we can check that they satisfy the complex-balanced conditions due to (13). Therefore, we prove the claim and thus $(G, \boldsymbol{k}, {\mathcal {I}}_0)$ is a complex-balanced system.

Second, we show that the input-output system $(G, \boldsymbol{k}, {\mathcal {I}}_0)$ exhibits infinitesimal homeostasis. From the construction, both the system $(G, \boldsymbol{k}, {\mathcal {I}})$ and the mass-action system $(\hat{G}, \hat{\boldsymbol{k}})$ share the same right-hand side except for the term involving $\frac{d x_1}{d t}$.

Moreover, the homeostasis matrix is derived from the Jacobian matrix by removing its row corresponding to $x_1$ and its column corresponding to $x_n$. Hence, we conclude that the homeostasis matrix *H* for the system $(G, \boldsymbol{k}, {\mathcal {I}})$ satisfies

14 $$\begin{aligned} \det (H) = \det (\hat{H}) = 0 \ \text { at } \ \boldsymbol{x}= \boldsymbol{x}^*. \end{aligned}$$

In the first part, we have shown that $\boldsymbol{x}^*$ is a complex-balanced equilibrium of the input-output system $(G, \boldsymbol{k}, {\mathcal {I}}_0)$ and it is non-degenerate since the stoichiometric subspace of *G* is $\mathcal {S}= \mathbb {R}^n$. Together with (14), we conclude that $(G, \boldsymbol{k}, {\mathcal {I}})$ exhibits infinitesimal homeostasis at ${\mathcal {I}}= {\mathcal {I}}_0$. $\square$

#### Remark 3.4

The system $(G, \boldsymbol{k}, {\mathcal {I}})$ shares the homeostasis matrix with the associated mass-action system $(\hat{G}, \hat{\boldsymbol{k}})$. However, $(G, \boldsymbol{k}, {\mathcal {I}})$ involves a varying input parameter ${\mathcal {I}}$, making it nontrivial to determine values of ${\mathcal {I}}$ for which the system becomes complex-balanced. In contrast, $(\hat{G}, \hat{\boldsymbol{k}})$ has fixed reaction rates and excludes both the vertex $\emptyset$ and the input parameter ${\mathcal {I}}$. As a result, verifying whether $(\hat{G}, \hat{\boldsymbol{k}})$ is complex-balanced and computing its homeostasis matrix becomes more straightforward.

#### Example 3.5

We revisit the input-output system $(G, \boldsymbol{k}, {\mathcal {I}})$ in Example 3.1, where *G* represents the E-graph in Figure 3, and all reaction rate constants are equal to 1, except for the rate of the inflow reaction ${\mathcal {I}}$.

**Fig. 4:**
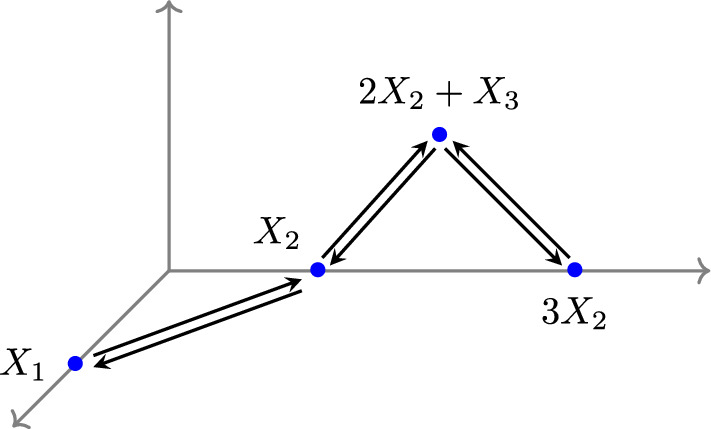
The mass-action system $(\hat{G}, \hat{\boldsymbol{k}})$ excludes the inflow reaction, with all reaction rate constants being equal to 1

Then we construct the associated mass-action system $(\hat{G}, \hat{\boldsymbol{k}})$ in Figure 4 and it satisfies

15 $$\begin{aligned} \begin{aligned}&\frac{d x_1}{dt} = - x_1 + x_2, \\ &\frac{d x_2}{dt} = x_1 - (x_2)^3, \\ &\frac{d x_3}{dt} = x_2 + (x_2)^3 - 2 (x_2)^2 x_3. \end{aligned} \end{aligned}$$

By direct computation, we verify that $\boldsymbol{x}^* = (1, 1, 1)$ is a complex-balanced equilibrium. Moreover, the homeostasis matrix of $(\hat{G}, \hat{\boldsymbol{k}})$ is given by

$$\begin{aligned} H = \begin{pmatrix} 1 & - 3 (x_2)^2 \\ 0 & 1 + 3 (x_2)^2 - 4 x_2 x_3 \end{pmatrix}, \end{aligned}$$

and thus

$$ \det (H) = 1 + 3 (x^*_2)^2 - 4 x^*_2 x^*_3 = 0 \ \text { at } \ \boldsymbol{x}^* = (1, 1, 1). $$

From Proposition 3.3, let ${\mathcal {I}}_0 = k_{X_1 \rightarrow \emptyset } (\boldsymbol{x}^*)^{X_1} = 1$. Then the input-output system $(G, \boldsymbol{k}, {\mathcal {I}})$ exhibits infinitesimal homeostasis at ${\mathcal {I}}= {\mathcal {I}}_0$ and $(G, \boldsymbol{k}, {\mathcal {I}}_0)$ is a complex-balanced system.

$$\square $$

In the remainder of this section, we establish a connection between infinitesimal homeostasis and a specific type of homeostasis, the null degradation type (see Remark 3.6 below).

#### Remark 3.6

( Golubitsky and Stewart (2017))

Recall from Theorem 2.15 and Definition 2.16 that the type of homeostasis may vary, depending on whether the determinant of certain homeostasis blocks $B_{\eta }$ in (6) is zero. In particular, when a homeostasis block $B_{\eta }$ is of size $1 \times 1$, it is referred to as a **Haldane** block if $B_{\eta } = f_{i,j}$ for $i \ne j$, and as a **null degradation** block if $B_{\eta } = f_{i,i}$.

#### Proposition 3.7

(Feinberg (1987))

Let $(G, \boldsymbol{k})$ be a complex-balanced system with a positive equilibrium $\boldsymbol{x}^* \in \mathbb {R}_{>0}^n$. Then it admits a cyclic decomposition, that is,

$$ (G, \boldsymbol{k}) = (G_1, \boldsymbol{k}_1) \ \bigoplus \ \cdots \ \bigoplus \ (G_p, \boldsymbol{k}_p), $$

where for every $1 \le i \le p$, $G_i$ is a cycle and $\boldsymbol{x}^*$ is a complex-balanced equilibrium of $(G_i, \boldsymbol{k}_i)$. $\square$

#### Lemma 3.8

Let $(G, \boldsymbol{k})$ be a mass-action system with the stoichiometric subspace $\mathcal {S}= \mathbb {R}^n$ given by

16 $$\begin{aligned} \frac{d \boldsymbol{x}}{d t} = \boldsymbol{f}(\boldsymbol{x}) = \sum _{\boldsymbol{y}_i \rightarrow \boldsymbol{y}_j \in E}k_{\boldsymbol{y}_i \rightarrow \boldsymbol{y}_j} \boldsymbol{x}^{\boldsymbol{y}_i}(\boldsymbol{y}_j - \boldsymbol{y}_i). \end{aligned}$$

Suppose $(G, \boldsymbol{k})$ has a complex-balanced equilibrium $\boldsymbol{x}^* \in \mathbb {R}_{>0}^n$, then for any $1 \le i \le n$,

$$ \frac{\partial f_i}{\partial x_i} \ne 0 \ \text { at } \ \boldsymbol{x}= \boldsymbol{x}^*, $$

where $\boldsymbol{f}(\boldsymbol{x}) = (f_1 (\boldsymbol{x}), \ldots , f_n (\boldsymbol{x}))$ is defined in (16).

#### Proof

We prove this lemma by contradiction. Suppose that $(G, \boldsymbol{k})$ has a complex-balanced equilibrium $\boldsymbol{x}^* \in \mathbb {R}_{>0}^n$, there exists $1 \le i \le n$ such that $\frac{\partial f_i}{\partial x_i} = 0$ at $\boldsymbol{x}= \boldsymbol{x}^*$. Without loss of generality, we assume that $i = 1$ and thus

$$ f_1 = \frac{\partial f_1}{\partial x_1} = 0 \ \text { at } \ \boldsymbol{x}= \boldsymbol{x}^*. $$

Since $(G, \boldsymbol{k})$ is a complex-balanced system, from Proposition 3.7 it can be decomposed into cycles $C_1, \ldots , C_p$ and $\boldsymbol{x}^*$ is a complex-balanced equilibrium for every cycle. Without loss of generality, we assume one of the cycles involved with $X_1$ as follows:

$$\begin{aligned} C_1: \boldsymbol{y}_1 \xrightarrow []{k_{1}} \boldsymbol{y}_2 \xrightarrow []{k_{2}} \boldsymbol{y}_3 \rightarrow \cdots \rightarrow \boldsymbol{y}_q \xrightarrow []{k_{q}} \boldsymbol{y}_1. \end{aligned}$$

where $\boldsymbol{y}_i \in V$ and at least one of $\{\boldsymbol{y}_i\}$ has $X_1$ component. Moreover, we have

17 $$\begin{aligned} k_{1} (\boldsymbol{x}^*)^{\boldsymbol{y}_1} = k_{2} (\boldsymbol{x}^*)^{\boldsymbol{y}_2} = \cdots = k_{q} (\boldsymbol{x}^*)^{\boldsymbol{y}_q}. \end{aligned}$$

Let $\Delta _i$ denote the difference of $X_1$ component on $\boldsymbol{y}_i$ and $\boldsymbol{y}_{i+1}$, that is,

18 $$\begin{aligned} \Delta _i = \boldsymbol{y}_{i+1, 1} - \boldsymbol{y}_{i, 1} \ \text { for } \ 1 \le i \le q - 1 \ \text { and } \ \Delta _q = \boldsymbol{y}_{1, 1} - \boldsymbol{y}_{q, 1}. \end{aligned}$$

Following the mass-action kinetics, we obtain the equation of the rate of change on $x_1$ in this cycle, given by

$$ \tilde{f}_1 = \sum \limits ^{q}_{i=1} k_{i} \boldsymbol{x}^{\boldsymbol{y}_i} \Delta _i. $$

From (18) applying the cycle property we have

$$ \sum \limits ^{n}_{i=1} \Delta _i = 0. $$

This, together with (17) implies

$$ \tilde{f}_1 = \sum \limits ^{q}_{i=1} k_{i} (\boldsymbol{x}^*)^{\boldsymbol{y}_i} \Delta _i = 0 \ \text { at } \ \boldsymbol{x}= \boldsymbol{x}^*. $$

Now, we take the partial derivative on $\tilde{f}_1$ getting

$$\begin{aligned} \frac{\partial \tilde{f}_1}{\partial x_1} = \sum \limits ^{q}_{i=1} k_{i} \boldsymbol{x}^{\boldsymbol{y}_i} \Delta _i \times \frac{\boldsymbol{y}_{i,1}}{x_1}. \end{aligned}$$

By substituting $\boldsymbol{x}= \boldsymbol{x}_{*}$, together with (17), we obtain

19 $$\begin{aligned} \frac{\partial \tilde{f}_1}{\partial x_1} (\boldsymbol{x}^*) = \sum \limits ^{q}_{i=1} k_{i} (\boldsymbol{x}^{*})^{\boldsymbol{y}_i} \Delta _i \times \frac{\boldsymbol{y}_{i,1}}{x^*_1} = \frac{k_{1} (\boldsymbol{x}^{*})^{\boldsymbol{y}_1}}{x^*_1} \sum \limits ^{q}_{i=1} \Delta _i \boldsymbol{y}_{i,1}. \end{aligned}$$

On the other hand, from (18) we have

$$\begin{aligned} \begin{aligned} \sum \limits ^{q}_{i=1} \Delta _i \boldsymbol{y}_{i,1}&= \sum \limits ^{q-1}_{i=1} (\boldsymbol{y}_{i+1, 1} - \boldsymbol{y}_{i, 1}) \boldsymbol{y}_{i,1} + (\boldsymbol{y}_{1, 1} - \boldsymbol{y}_{q, 1}) \boldsymbol{y}_{q,1} \\ &= - \frac{1}{2} \sum \limits ^{q-1}_{i=1} (\boldsymbol{y}_{i+1, 1} - \boldsymbol{y}_{i, 1})^2 - \frac{1}{2} (\boldsymbol{y}_{1, 1} - \boldsymbol{y}_{q, 1})^2 \le 0, \end{aligned} \end{aligned}$$

where the equality holds when $\boldsymbol{y}_{1,1} = \ldots = \boldsymbol{y}_{q,1}$. Thus, $\frac{\partial \tilde{f}_1}{\partial x_1} (\boldsymbol{x}^*) = 0$ holds when all vertices in the cycle have the same amount of $X_1$, which indicates $\tilde{f}_1 \equiv 0$.

Analogously, we can deduce a similar result for the rest of the decomposed cycles $C_2, \ldots , C_p$. For any $1 \le 2 \le p$, $\frac{\partial \tilde{f}_i}{\partial x_i} (\boldsymbol{x}^*) = 0$ holds when all vertices in the cycle $C_i$ have the same amount of $X_1$, indicating $\tilde{f}_i \equiv 0$. Therefore, $\frac{\partial f_1}{\partial x_1} (\boldsymbol{x}^*) = 0$ holds when all vertices in the cycle have the same amount of $X_1$ and thus

$$ f_1 = \sum \tilde{f}_1 + \ldots \tilde{f}_p \equiv 0. $$

However, this contradicts our assumption that the stoichiometric subspace $\mathcal {S}= \mathbb {R}^n$ $\square$

Recall the notion of null degradation defined in Remark 3.6. The following remark follows from Lemma 3.8 and Deficiency Zero Theorem (Theorem 2.9).

#### Remark 3.9

Assume that the input–output system $(G, \boldsymbol{k}, {\mathcal {I}})$ has stoichiometric subspace $\mathcal {S}= \mathbb {R}^n$.

- If the input-output system $(G, \boldsymbol{k}, {\mathcal {I}})$ exhibits infinitesimal homeostasis at ${\mathcal {I}}_0$ and $(G, \boldsymbol{k}, {\mathcal {I}}_0)$ is a complex-balanced system, then the infinitesimal homeostasis at ${\mathcal {I}}_0$ is not of the null degradation type.
- Suppose the input-output system $(G, \boldsymbol{k}, {\mathcal {I}})$ is weakly reversible and has zero deficiency. If the system exhibits infinitesimal homeostasis at ${\mathcal {I}}_0$, then it is not of the null degradation type.

## Main results

Now we present the main results of this paper, providing an explicit method to compute *infinitesimal homeostasis* and *infinitesimal concentration robustness* on E-graphs with $\mathcal {S}\subsetneq \mathbb {R}^n$. The following settings for mass-action systems will be used throughout this section.

Consider the mass-action system $(G, \boldsymbol{k})$ as follows:

20 $$\begin{aligned} \frac{d \boldsymbol{x}}{d t} = \boldsymbol{f}(\boldsymbol{x}) = \sum _{\boldsymbol{y}\rightarrow \boldsymbol{y}' \in E}k_{\boldsymbol{y}\rightarrow \boldsymbol{y}'} \boldsymbol{x}^{\boldsymbol{y}}(\boldsymbol{y}' - \boldsymbol{y}), \end{aligned}$$

where $\boldsymbol{f}= (f_1, \ldots , f_n)$. Assume that its stoichiometric subspace $\mathcal {S}\subsetneq \mathbb {R}^n$ satisfies

21 $$\begin{aligned} \dim (\mathcal {S}) = n - d < n \ \text { and } \ \dim (\mathcal {S}^{\perp }) = d > 0, \end{aligned}$$

Recall from Remark 2.13 that the function $f_n$ in (20) is assumed to be not identically zero. Since the remaining functions $f_1, \ldots , f_{n-1}$ on the right-hand side of (20) can be reordered, we may, without loss of generality, assume that the $\{ f_{d + 1}, \ldots , f_{n} \}$ are linearly independent and

22 $$\begin{aligned} f_i \in {{\,\textrm{span}\,}}\{ f_{d + 1}, \ldots , f_{n} \} \ \text { for } \ 1 \le i \le d. \end{aligned}$$

Further, denote an orthonormal basis of $\mathcal {S}^{\perp }$ by

23 $$\begin{aligned} U = \{ \boldsymbol{u}_1, \ldots , \boldsymbol{u}_d \} \subset \mathbb {R}^n, \end{aligned}$$

where $\boldsymbol{u}_i = (\boldsymbol{u}_{i, 1}, \ldots , \boldsymbol{u}_{i, n})^{\intercal }$ for $1 \le i \le d$. Note that from (2) it follows that $(G, \boldsymbol{k})$ has linear conservation laws, such that

24 $$\begin{aligned} \boldsymbol{u}_i \cdot \frac{d \boldsymbol{x}}{d t} = 0 \ \text { for } \ 1 \le i \le d. \end{aligned}$$

$$\square $$

### Remark 4.1

Note that, for computational convenience, we chose to work with an orthonormal basis in the above proof. However, the assumption of orthonormality for the basis of $\mathcal {S}^{\perp }$ does not constrain the subsequent analysis.

### Modified jacobian matrix

In Section 3, we focused on mass-action systems with $\mathcal {S}= \mathbb {R}^n$, where infinitesimal homeostasis is well-defined around a non-degenerate equilibrium.

However, the Jacobian matrix is singular when $\mathcal {S}\subsetneq \mathbb {R}^n$, indicating that the system cannot guarantee a non-degenerate equilibrium. Therefore, we introduce the *Jacobian matrix of the “extended rate function”* (Feliu and Wiuf 2012), which plays a crucial role in establishing our results on infinitesimal homeostasis and infinitesimal concentration robustness. For better clarity, we refer to it throughout this paper as the *modified Jacobian matrix* and give its formal definition below.

#### Definition 4.2

( Feliu and Wiuf (2012))

Let $(G, \boldsymbol{k})$ be a mass-action system as defined in (20). Suppose it satisfies (21)-(23) and the conservation laws (24). Define the **modified Jacobian matrix** $\tilde{J}$ associated with the system $(G, \boldsymbol{k})$ as

25 $$\begin{aligned} \tilde{J} = \begin{pmatrix} \boldsymbol{u}_{1, 1} & \cdots & \boldsymbol{u}_{1, n} \\ \vdots & \ddots & \vdots \\ \boldsymbol{u}_{d, 1} & \cdots & \boldsymbol{u}_{d, n} \\ \frac{\partial f_{d+1}}{\partial x_1} & \cdots & \frac{\partial f_{d+1}}{\partial x_n} \\ \vdots & \ddots & \vdots \\ \frac{\partial f_{n}}{\partial x_1} & \cdots & \frac{\partial f_{n}}{\partial x_n} \end{pmatrix}. \end{aligned}$$

Further, for each $1 \le i \le n$, let $\tilde{H}_i$ denote the $i^{th}$ **modified homeostasis matrix** obtained by removing the $i^{th}$ row and the $n^{th}$ column from $\tilde{J}$.

#### Definition 4.3

( Craciun and Feinberg (2010); Banaji and Pantea (2016))

Let $(G, \boldsymbol{k})$ be a mass-action system as defined in (20). Suppose it satisfies (21)-(23) and the conservation laws (24). Let *J* denote the Jacobian matrix of $(G, \boldsymbol{k})$. An equilibrium $\boldsymbol{x}^* \in \mathbb {R}_{>0}^n$ is called a **non-degenerate equilibrium relative to its stoichiometric compatibility class** of $(G, \boldsymbol{k})$ if

26 $$\begin{aligned} \ker (J) \cap \mathcal {S}= \{ \textbf{0} \} \ \text { at } \ \boldsymbol{x}= \boldsymbol{x}^*. \end{aligned}$$

For simplicity, we refer to such an equilibrium as a **non-degenerate equilibrium** in the rest of this paper.

Suppose the mass-action system $(G, \boldsymbol{k})$ in (20) satisfies (21)-(23) and the conservation laws (24). From (20) and (22), we assume that27 Let us fix a stoichiometric compatibility class $\mathcal {S}_{\boldsymbol{x}_0}$. Consider any solution $\boldsymbol{x}(t) = (x_1 (t), \ldots , x_n (t)) \in \mathcal {S}_{\boldsymbol{x}_0}$ of the system $(G, \boldsymbol{k})$. The conservation laws (24) and (27) imply that there exist constants $c_1, \ldots , c_{n-d}$, such that

28 $$\begin{aligned} x_i = \sum \limits ^{n}_{j=d+1} a_{i,j} x_j + c_i \ \text { for } \ 1 \le i \le d. \end{aligned}$$

In view of (28), we may write

$$ \tilde{f}_{j} (x_{d+1}, \ldots , x_n) = f_{j} (x_1, \ldots , x_n) \ \text { for } \ 1 \le j \le d. $$

Define the **modified mass-action system** associated with $(G, \boldsymbol{k})$ as follows:29 Assume $(G, \boldsymbol{k})$ has an equilibrium at $\boldsymbol{x}= \boldsymbol{x}^* \in \mathcal {S}_{\boldsymbol{x}_0}$. Note that $\boldsymbol{x}^*$ is also an equilibrium of the modified mass-action system (29).

The following proposition establishes the connection between a non-degenerate equilibrium and the modified mass-action system.

#### Proposition 4.4

Consider the mass-action system $(G, \boldsymbol{k})$ in (20). Suppose it satisfies (21)-(23) and the conservation laws (24). Then $(G, \boldsymbol{k})$ has a non-degenerate equilibrium $\boldsymbol{x}^*$ if and only if the Jacobian matrix of the modified mass-action system associated with $(G, \boldsymbol{k})$ is non-singular at $\boldsymbol{x}= \boldsymbol{x}^*$, that is,

30 $$\begin{aligned} \det \begin{pmatrix} \frac{\partial \tilde{f}_{d + 1}}{\partial x_{d + 1}} & \cdots & \frac{\partial \tilde{f}_{d + 1}}{\partial x_n} \\ \vdots & \ddots & \vdots \\ \frac{\partial \tilde{f}_{n}}{\partial x_{d + 1}} & \cdots & \frac{\partial \tilde{f}_{n}}{\partial x_n} \end{pmatrix} \ne 0 \ \text { at } \ \boldsymbol{x}= \boldsymbol{x}^*. \end{aligned}$$

#### Proof

Since the mass-action system $(G, \boldsymbol{k})$ satisfies (22), then

$$\begin{aligned} f_i \in {{\,\textrm{span}\,}}\{ f_{d+1}, \ldots , f_{n} \} \ \text { for } \ 1 \le i \le d. \end{aligned}$$

For the state $\boldsymbol{x}^*$, consider the stoichiometric compatibility class

$$ \mathcal {S}_{\boldsymbol{x}^*} = (\boldsymbol{x}^* + \mathcal {S}) \cap \mathbb {R}_{>0}^n. $$

Without loss of generality, assume (27) and (28), and construct the modified mass-action system (29). Then it satisfies

31 $$\begin{aligned} \frac{\partial \tilde{f}_{j}}{\partial x_{i}} = \frac{\partial f_{j}}{\partial x_{1}} a_{1, i} + \cdots + \frac{\partial f_{j}}{\partial x_{d}} a_{d, i} + \frac{\partial f_{j}}{\partial x_{i}}. \end{aligned}$$

$(\implies )$ First, suppose that $(G, \boldsymbol{k})$ has a non-degenerate equilibrium $\boldsymbol{x}^*$. We proceed by contradiction: assume that the Jacobian matrix of the modified mass-action system associated with $(G, \boldsymbol{k})$ is singular at $\boldsymbol{x}= \boldsymbol{x}^*$. Then there exists a non-zero vector $(v_{d+1}, \ldots , v_n)^{\intercal }$ such that

32 $$\begin{aligned} \begin{pmatrix} \frac{\partial \tilde{f}_{d + 1}}{\partial x_{d + 1}} & \cdots & \frac{\partial \tilde{f}_{d + 1}}{\partial x_n} \\ \vdots & \ddots & \vdots \\ \frac{\partial \tilde{f}_{n}}{\partial x_{d + 1}} & \cdots & \frac{\partial \tilde{f}_{n}}{\partial x_n} \end{pmatrix} \begin{pmatrix} v_{d+1} \\ \vdots \\ v_n \end{pmatrix} = \textbf{0} \ \text { at } \ \boldsymbol{x}= \boldsymbol{x}^*. \end{aligned}$$

Denote a vector $\boldsymbol{v}= (v_{1}, \ldots , v_n)^{\intercal }$ as

33 $$\begin{aligned} \begin{pmatrix} v_{1} \\ \vdots \\ v_d \end{pmatrix} = \begin{pmatrix} a_{1, d+1} & \cdots & a_{1, n} \\ \vdots & \ddots & \vdots \\ a_{d, d+1} & \cdots & a_{d, n} \end{pmatrix} \begin{pmatrix} v_{d+1} \\ \vdots \\ v_n \end{pmatrix}. \end{aligned}$$

The assumption (27) implies that $\boldsymbol{v}\in \mathcal {S}$. Combining (31), (32) and (33), we derive that

$$\begin{aligned} \begin{pmatrix} \frac{\partial f_{1}}{\partial x_{1}} & \cdots & \frac{\partial f_{1}}{\partial x_n} \\ \vdots & \ddots & \vdots \\ \frac{\partial f_{n}}{\partial x_{1}} & \cdots & \frac{\partial f_{n}}{\partial x_n} \end{pmatrix} \begin{pmatrix} v_1 \\ \vdots \\ v_n \end{pmatrix} = \textbf{0} \ \text { at } \ \boldsymbol{x}= \boldsymbol{x}^*, \end{aligned}$$

and thus

$$ \boldsymbol{v}\in \ker (J) \cap \mathcal {S}\ \text { at } \ \boldsymbol{x}= \boldsymbol{x}^*. $$

This contradicts the assumption that $\boldsymbol{x}^*$ is a non-degenerate equilibrium of $(G, \boldsymbol{k})$.

 Second, suppose that the Jacobian matrix of the modified mass-action system associated with $(G, \boldsymbol{k})$ is non-singular at $\boldsymbol{x}= \boldsymbol{x}^*$. We proceed by contradiction: assume that $\boldsymbol{x}^*$ is not a non-degenerate equilibrium of $(G, \boldsymbol{k})$. Then there exists a non-zero vector $\boldsymbol{v}= (v_1, \ldots , v_n)^{\intercal }$, such that

$$\begin{aligned} \boldsymbol{v}\in \ker (J) \cap \mathcal {S}\ \text { at } \ \boldsymbol{x}= \boldsymbol{x}^*. \end{aligned}$$

Since $\boldsymbol{v}\in \mathcal {S}$, the assumption (27) implies

$$\begin{aligned} v_i = \sum \limits ^{n}_{j=d+1} a_{i,j} v_j \ \text { for } \ 1 \le i \le d. \end{aligned}$$

This, together with (31), shows that

$$\begin{aligned} \begin{pmatrix} \frac{\partial \tilde{f}_{d + 1}}{\partial x_{d + 1}} & \cdots & \frac{\partial \tilde{f}_{d + 1}}{\partial x_n} \\ \vdots & \ddots & \vdots \\ \frac{\partial \tilde{f}_{n}}{\partial x_{d + 1}} & \cdots & \frac{\partial \tilde{f}_{n}}{\partial x_n} \end{pmatrix} \begin{pmatrix} v_{d+1} \\ \vdots \\ v_n \end{pmatrix} = \textbf{0} \ \text { at } \ \boldsymbol{x}= \boldsymbol{x}^*. \end{aligned}$$

Since the Jacobian matrix of the modified mass-action system associated with $(G, \boldsymbol{k})$ is non-singular at $\boldsymbol{x}= \boldsymbol{x}^*$, we conclude that $v_{d+1} = \cdots = v_n = 0$. This further implies that $\boldsymbol{v}= \textbf{0}$, which contradicts the assumption that $\boldsymbol{v}$ is nonzero. $\square$

#### Lemma 4.5

Consider the mass-action system $(G, \boldsymbol{k})$ in (20). Suppose it satisfies (21)-(23) and the conservation laws (24). Then $(G, \boldsymbol{k})$ has a non-degenerate equilibrium $\boldsymbol{x}= \boldsymbol{x}^*$ if and only if

34 $$\begin{aligned} \det (\tilde{J}) \ne 0 \ \text { at } \ \boldsymbol{x}= \boldsymbol{x}^*, \end{aligned}$$

where $\tilde{J}$ is the modified Jacobian matrix defined in (25).

#### Proof

$(\implies )$ First, assume that $(G, \boldsymbol{k})$ has a non-degenerate equilibrium $\boldsymbol{x}^*$. Then the assumption (22) shows

35 $$\begin{aligned} \ker \begin{pmatrix} \frac{\partial f_{d+1}}{\partial x_1} & \cdots & \frac{\partial f_{d+1}}{\partial x_n} \\ \vdots & \ddots & \vdots \\ \frac{\partial f_{n}}{\partial x_1} & \cdots & \frac{\partial f_{n}}{\partial x_n} \end{pmatrix} \cap \mathcal {S}= \{ \textbf{0} \} \ \text { at } \ \boldsymbol{x}= \boldsymbol{x}^*. \end{aligned}$$

Suppose (34) fails, then there exists a non-zero vector $\boldsymbol{v}$ such that

$$ \textbf{0} \ne \boldsymbol{v}\in \ker (\tilde{J}) \ \text { at } \ \boldsymbol{x}= \boldsymbol{x}^*. $$

From (35), we get $\boldsymbol{v}\notin \mathcal {S}$ and thus it can be expressed as

$$\begin{aligned} \boldsymbol{v}= \boldsymbol{v}_1 + \boldsymbol{v}_2 \ \text { with } \ \boldsymbol{v}_1 \in \mathcal {S}\ \text { and } \ \textbf{0} \ne \boldsymbol{v}_2 \in \mathcal {S}^{\perp }. \end{aligned}$$

Together with the assumption (23) that $\{\boldsymbol{u}_1, \ldots , \boldsymbol{u}_d \}$ is a basis of $\mathcal {S}^{\perp }$, we obtain

$$\begin{aligned} \begin{pmatrix} {\boldsymbol{u}}_{1, 1} & \cdots & {\boldsymbol{u}}_{1, n} \\ \vdots & \ddots & \vdots \\ {\boldsymbol{u}}_{d, 1} & \cdots & {\boldsymbol{u}}_{d, n} \end{pmatrix} \cdot {\boldsymbol{v}} = \begin{pmatrix} {\boldsymbol{u}}_{1, 1} & \cdots & {\boldsymbol{u}}_{1, n} \\ \vdots & \ddots & \vdots \\ {\boldsymbol{u}}_{d, 1} & \cdots & {\boldsymbol{u}}_{d, n} \end{pmatrix} \cdot {\boldsymbol{v}}_2 \ne {\boldsymbol{0}}. \end{aligned}$$

This contradicts the assumption that ${\boldsymbol{v}} \in \ker (\tilde{J})$, and hence (34) holds.

 Second, assume that (34) holds. Suppose that $\boldsymbol{x}^*$ is a degenerate equilibrium of $(G, \boldsymbol{k})$. This implies that there exists a non-zero vector $\boldsymbol{v}$ such that

$$\begin{aligned} \boldsymbol{v}\in \ker \begin{pmatrix} \frac{\partial f_{d+1}}{\partial x_1} & \cdots & \frac{\partial f_{d+1}}{\partial x_n} \\ \vdots & \ddots & \vdots \\ \frac{\partial f_{n}}{\partial x_1} & \cdots & \frac{\partial f_{n}}{\partial x_n} \end{pmatrix} \cap \mathcal {S}\ \text { at } \ \boldsymbol{x}= \boldsymbol{x}^*. \end{aligned}$$

Since $\boldsymbol{v}\in \mathcal {S}$, the definition of the modified Jacobian matrix $\tilde{J}$ in (25) implies that

$$ \boldsymbol{v}\in \ker (\tilde{J}) \ \text { at } \ \boldsymbol{x}= \boldsymbol{x}^*. $$

This contradicts the assumption (34), and we complete the proof. $\square$

### Deterministic systems

In what follows, it will be convenient to consider the mass-action system (20) as a general input-output system $(G, \boldsymbol{k}, {\mathcal {I}})$ where now the input parameter ${\mathcal {I}}$ may take a more general form. First, let us suppose that ${{\mathcal {I}}}=k_{ij}$ is a *reaction rate constant associated with the edge* $\boldsymbol{y}_i \rightarrow \boldsymbol{y}_{j} \in E$, given by

36 $$\begin{aligned} \boldsymbol{y}_{i} \xrightarrow {k_{ij}} \boldsymbol{y}_{j}. \end{aligned}$$

Suppose that the input parameter $k_{ij}$ appears only in one of the equations in (20). Under mass-action kinetics, we further assume

$$ \boldsymbol{y}_j - \boldsymbol{y}_i \in {{\,\textrm{span}\,}}\{ \textbf{e}_p \} \ \text { for some } p. $$

This implies that $\textbf{e}_p \in \mathcal {S}$. If $p \ne n$, the two functions $f_p$ and $f_n$ on the right-hand side of (20) are linearly independent. Since the functions $f_i$ on the right-hand side can be reordered, in addition to the assumption in (22), we may, without loss of generality, assume that $d + 1 \le p \le n$. Furthermore, $x_n$ is the output parameter, and every reaction in $(G, \boldsymbol{k})$ except the reaction (36) has a fixed reaction rate constant.

#### Definition 4.6

Let $(G, \boldsymbol{k})$ be a mass-action system as defined in (20). Suppose the input-output function $x_n (k_{ij})$ is well-defined in a neighborhood of $k^*_{ij}$. **Infinitesimal homeostasis** occurs at $k^*_{ij}$ if

37 $$\begin{aligned} \frac{d}{d k_{ij}} x_n (k^*_{ij}) = 0. \end{aligned}$$

The following is our main theorem about infinitesimal homeostasis in mass-action systems with $\mathcal {S}\subsetneq \mathbb {R}^n$.

#### Theorem 4.7

Consider the mass-action system $(G, \boldsymbol{k})$ in (20) with stoichiometric subspace $\mathcal {S}\subsetneq \mathbb {R}^n$. Suppose it satisfies (21)-(24) and includes the reaction in (36). Let $\boldsymbol{x}_0 \in \mathbb {R}_{>0}^n$, assume the system has a non-degenerate equilibrium $\boldsymbol{x}= \boldsymbol{x}^* \in \big ( \boldsymbol{x}_0 + \mathcal {S}\big ) \cap \mathbb {R}^n_{>0}$ when $k_{ij} = k_{ij}^*$, and

$$\begin{aligned} \boldsymbol{y}_j - \boldsymbol{y}_i \in {{\,\textrm{span}\,}}\{ \textbf{e}_p \} \ \text { for some } d + 1 \le p \le n. \end{aligned}$$

We say that $(G, \boldsymbol{k})$ exhibits infinitesimal homeostasis at $k^*_{ij}$ if and only if

$$\begin{aligned} \det ( \tilde{H_{p}} ) = 0 \ \text { at } \ \boldsymbol{x}= \boldsymbol{x}^*, \end{aligned}$$

where $\tilde{H}_p$ is the $p^{th}$ modified homeostasis matrix defined in Definition 4.2.

#### Proof

Under the mass-action system (20), we get

$$\begin{aligned} \frac{d \boldsymbol{x}}{d t} = \boldsymbol{f}(\boldsymbol{x}) = \sum _{\boldsymbol{y}\rightarrow \boldsymbol{y}' \in E}k_{\boldsymbol{y}\rightarrow \boldsymbol{y}'} \boldsymbol{x}^{\boldsymbol{y}}(\boldsymbol{y}' - \boldsymbol{y}), \end{aligned}$$

where $\boldsymbol{f}(\boldsymbol{x}) = (f_1 (\boldsymbol{x}), \ldots , f_n (\boldsymbol{x}))^{\intercal }$. Since $\boldsymbol{y}_i \rightarrow \boldsymbol{y}_j \in E$ and $\boldsymbol{y}_j - \boldsymbol{y}_i \in {{\,\textrm{span}\,}}\{ \textbf{e}_p \}$ for some $d + 1 \le p \le n$, there exists a non-zero integer $c \in \mathbb {Z}\backslash \{ 0 \}$, such that

$$\begin{aligned} \boldsymbol{y}_j - \boldsymbol{y}_i = c \times \textbf{e}_p. \end{aligned}$$

This implies that $k_{ij}$ only appears in $f_p (\boldsymbol{x})$, that is,

38 $$\begin{aligned} \frac{d}{d k_{ij} } f_{\ell } (\boldsymbol{x}) = {\left\{ \begin{array}{ll} c\, \boldsymbol{x}^{\boldsymbol{y}_i}, & \ell = p, \\ 0, & \ell \ne p. \end{array}\right. } \end{aligned}$$

On the other hand, the assumptions (22) and (24) indicate that the mass-action system $(G, \boldsymbol{k})$ having an equilibrium $\boldsymbol{x}= \boldsymbol{x}^*$ is equivalent to solving the following system:

39 $$\begin{aligned} \begin{aligned}&f_{d+1} (\boldsymbol{x}) = 0, \ldots , f_n (\boldsymbol{x}) = 0, \\ &\boldsymbol{u}_1 \cdot \boldsymbol{x}= \boldsymbol{u}_1 \cdot \boldsymbol{x}_0, \ldots , \boldsymbol{u}_d \cdot \boldsymbol{x}= \boldsymbol{u}_d \cdot \boldsymbol{x}_0. \end{aligned} \end{aligned}$$

We can compute that $\boldsymbol{x}= \boldsymbol{x}^*$ is an equilibrium of (39) and the corresponding Jacobian matrix is the modified Jacobian matrix $\tilde{J}$ defined in (25), that is,

$$ \tilde{J} = \begin{pmatrix} \boldsymbol{u}_{1, 1} & \cdots & \boldsymbol{u}_{1, n} \\ \vdots & \ddots & \vdots \\ \boldsymbol{u}_{d, 1} & \cdots & \boldsymbol{u}_{d, n} \\ \frac{\partial f_{d+1}}{\partial x_1} & \cdots & \frac{\partial f_{d+1}}{\partial x_n} \\ \vdots & \ddots & \vdots \\ \frac{\partial f_{n}}{\partial x_1} & \cdots & \frac{\partial f_{n}}{\partial x_n} \end{pmatrix}. $$

Moreover, since $\boldsymbol{x}= \boldsymbol{x}^*$ is a non-degenerate equilibrium, Lemma 4.5 shows that $\tilde{J}$ is non-singular at $\boldsymbol{x}= \boldsymbol{x}^*$. Thus, the implicit function theorem implies the existence of an open interval containing $k_{ij}$, that is,

$$ U = (k^*_{ij} - \varepsilon , k^*_{ij} + \varepsilon ), $$

such that there exists a unique and smooth family of equilibria

40 $$\begin{aligned} \boldsymbol{x}= (x_1 (k_{ij}), \ldots , x_n (k_{ij})), \end{aligned}$$

solving (39) for every $k_{ij} \in U$. Taking the derivative with respect to $k_{ij}$ on the system (39), together with (38) and (40), we obtain thatwhere $\prime$ indicates differentiation with respect to $k_{ij}$. Recall that $\boldsymbol{x}^* \in \mathbb {R}^n_{>0}$ and $c \in \mathbb {Z}\backslash \{ 0 \}$, then

$$\begin{aligned} c \boldsymbol{x}^{\boldsymbol{y}_i} \ne 0. \end{aligned}$$

By Cramer’s rule, we obtain that

$$ x^{\prime }_n (k_{ij}) = - \frac{c \boldsymbol{x}^{\boldsymbol{y}_i}}{\det (\tilde{J} ) } \det (\tilde{H}_p), $$

and thus complete the proof of the theorem. $\square$

#### Remark 4.8

We remark that the assumption $y_j-y_i \in {{\,\textrm{span}\,}}\{ e_p \}$ ensures that the input rate constant influences the rate of a reaction that modifies only one species directly. As a result, the analysis of infinitesimal homeostasis in Theorem 4.7 involves a single determinant of the associated homeostasis matrix.

In contrast, if the reaction affects multiple species simultaneously−that is, if $\boldsymbol{y}_j - \boldsymbol{y}_i$ has support on more than one coordinate−then the condition for infinitesimal homeostasis generally involves a linear combination of multiple minors of the Jacobian matrix. Such cases lie beyond the scope of the present work, as verifying homeostasis in these settings requires evaluating multiple determinants of the associated homeostasis matrices (see, e.g., Appendix E in Yu and Sontag (2024) and the discussion therein).

The following results are consequences of Theorem 4.7 and the Deficiency Zero Theorem (Theorem 2.9).

#### Corollary 4.9

Consider the mass-action system $(G, \boldsymbol{k})$ in (20) with stoichiometric subspace $\mathcal {S}\subsetneq \mathbb {R}^n$. Suppose it satisfies (21)-(24) and includes the reaction in (36). Let $\boldsymbol{x}_0 \in \mathbb {R}_{>0}^n$, assume the system has a complex-balanced equilibrium $\boldsymbol{x}= \boldsymbol{x}^* \in \big ( \boldsymbol{x}_0 + \mathcal {S}\big ) \cap \mathbb {R}^n_{>0}$ when $k_{ij} = k_{ij}^*$, and

$$\begin{aligned} \boldsymbol{y}_j - \boldsymbol{y}_i \in {{\,\textrm{span}\,}}\{ \textbf{e}_p \} \ \text { for some } d + 1 \le p \le n. \end{aligned}$$

Then $(G, \boldsymbol{k})$ exhibits infinitesimal homeostasis at $k^*_{ij}$ if and only if the $p^{th}$ modified homeostasis matrix satisfies

$$\begin{aligned} \det ( \tilde{H_{p}} ) = 0 \ \text { at } \ \boldsymbol{x}= \boldsymbol{x}^*. \end{aligned}$$

#### Proof

From Theorem 2.7, every complex-balanced equilibrium is linearly stable in each stoichiometric compatibility class. Further, Craciun et al. (2023) shows that the modified Jacobian matrix at every complex-balanced equilibrium is non-singular. Therefore, we conclude this corollary from Theorem 4.7. $\square$

#### Corollary 4.10

Let *G* be a weakly reversible and deficiency zero E-graph, and let $(G, \boldsymbol{k})$ be a mass-action system in (20) with stoichiometric subspace $\mathcal {S}\subsetneq \mathbb {R}^n$. Suppose $(G, \boldsymbol{k})$ satisfies (21)-(24) and includes the reaction in (36). Let $\boldsymbol{x}_0 \in \mathbb {R}_{>0}^n$, assume $(G, \boldsymbol{k})$ has a positive equilibrium $\boldsymbol{x}= \boldsymbol{x}^* \in \big ( \boldsymbol{x}_0 + \mathcal {S}\big ) \cap \mathbb {R}^n_{>0}$ when $k_{ij} = k_{ij}^*$, and

$$\begin{aligned} \boldsymbol{y}_j - \boldsymbol{y}_i \in {{\,\textrm{span}\,}}\{ \textbf{e}_p \} \ \text { for some } d + 1 \le p \le n. \end{aligned}$$

Then $(G, \boldsymbol{k})$ exhibits infinitesimal homeostasis at $k^*_{ij}$ if and only if the $p^{th}$ modified homeostasis matrix satisfies

$$\begin{aligned} \det ( \tilde{H_{p}} ) = 0 \ \text { at } \ \boldsymbol{x}= \boldsymbol{x}^*. \end{aligned}$$

#### Proof

From Theorem 2.9, $(G, \boldsymbol{k})$ is a complex-balanced system for any reaction rate vector $\boldsymbol{k}\in \mathbb {R}^{|E|}_{>0}$, and thus $\boldsymbol{x}= \boldsymbol{x}^*$ is a complex-balanced equilibrium. This corollary directly follows from Corollary 4.9. $\square$

Up to this point, given the mass-action system $(G, \boldsymbol{k})$ in (20) and the associated input-output system $(G, \boldsymbol{k}, {\mathcal {I}})$, we have consistently chosen ${\mathcal {I}}$ to represent a reaction rate constant. We will now modify this approach as follows. Suppose $(G, \boldsymbol{k})$ satisfies (21)-(24) and the conservation laws are given by

$$\begin{aligned} \boldsymbol{u}_i \cdot \boldsymbol{x}(t) \equiv M_i \ \text { for } \ i = 1, \ldots , d. \end{aligned}$$

Let the input parameter ${\mathcal {I}}$ be the conservation law constant $M_i$ (denoted by ${\mathcal {I}}= M_i$), and let $x_n$ be the output parameter. Note that all reaction rate constants in $(G, \boldsymbol{k})$ are fixed. In the following, we define the notion of *infinitesimal concentration robustness*.

#### Definition 4.11

Let $(G, \boldsymbol{k})$ be a mass-action system as defined in (20). Suppose it satisfies (21)-(24) and the conservation laws

$$\begin{aligned} \boldsymbol{u}_i \cdot \boldsymbol{x}(t) \equiv M_i \ \text { for } \ i = 1, \ldots , d. \end{aligned}$$

For any $1 \le i \le d$, suppose the input-output function $x_n (M_{i})$ is well-defined in a neighborhood of $M^*_{i}$. The $i^{th}$ **infinitesimal concentration robustness** occurs at $M^*_{i}$ if

41 $$\begin{aligned} \frac{d}{d M_{i}} x_n (M^*_{i}) = 0. \end{aligned}$$

Moreover, suppose the input-output function $x_n (M_{i})$ is well-defined in a neighborhood of $M^*_{i}$ for every $1 \le i \le d$. **Complete infinitesimal concentration robustness** occurs at $M^* = (M^*_{1}, \ldots , M^*_{d})$, if $i^{th}$ infinitesimal concentration robustness occurs at $M^*_{i}$ for every $1 \le i \le d$.

#### Remark 4.12

The notion of *infinitesimal concentration robustness* is closely related to the concept of *absolute concentration robustness* (ACR), introduced in Shinar and Feinberg (2010). While ACR requires that the steady-state concentration of a species remains exactly constant across all admissible initial conditions, infinitesimal concentration robustness pertains to the local behavior of the input-output function. In particular, it is characterized by local flatness of the trajectory near equilibrium and does not imply invariance throughout the entire set of initial conditions.

Now we present our main result regarding infinitesimal concentration robustness in mass-action systems with $\mathcal {S}\subsetneq \mathbb {R}^n$.

#### Theorem 4.13

Consider the mass-action system $(G, \boldsymbol{k})$ in (20). Suppose $(G, \boldsymbol{k})$ satisfies (21)-(24) and the conservation laws are given by

$$\begin{aligned} \boldsymbol{u}_i \cdot \boldsymbol{x}(t) \equiv M_i \ \text { for } \ i = 1, \ldots , d. \end{aligned}$$

Assume $(G, \boldsymbol{k})$ has a non-degenerate equilibrium $\boldsymbol{x}= \boldsymbol{x}^* \in \mathbb {R}^n_{>0}$ when $M = (M^*_{1}, \ldots , M^*_{d})$. Then for any $1 \le i \le d$, $(G, \boldsymbol{k})$ exhibits the $i^{th}$ infinitesimal concentration robustness at $M^*_{i}$ if and only if

42 $$\begin{aligned} \det ( \tilde{H}_{i} ) = 0 \ \text { at } \ \boldsymbol{x}= \boldsymbol{x}^*, \end{aligned}$$

where $\tilde{H}_i$ is the $i^{th}$ modified homeostasis matrix defined in Definition 4.2. Consequentially, $(G, \boldsymbol{k})$ exhibits complete infinitesimal concentration robustness at $(M^*_{1}, \ldots , M^*_{d})$, if and only if (42) holds for every $1 \le i \le d$.

#### Proof

Without loss of generality, we assume $i = 1$ and thus the input parameter is $M_1$. Under the mass-action system (20), we get

$$\begin{aligned} \frac{ d \boldsymbol{x}}{ dt } = {\boldsymbol{f}} (\boldsymbol{x}) = \sum _{\boldsymbol{y}_i \rightarrow \boldsymbol{y}_j \in E} k_{ij} \boldsymbol{x}^{\boldsymbol{y}_i} (\boldsymbol{y}_j - \boldsymbol{y}_i). \end{aligned}$$

From (22) and (24), $(G, \boldsymbol{k})$ having an equilibrium $\boldsymbol{x}= \boldsymbol{x}^*$ when $M = (M^*_{1}, \ldots , M^*_{d})$ is equivalent to solving the following system:

43 $$\begin{aligned} \begin{aligned}&f_{d+1} (\boldsymbol{x}) = 0, \ldots , f_n (\boldsymbol{x}) = 0, \\ &\boldsymbol{u}_1 \cdot \boldsymbol{x}= M^*_{1}, \ldots , \boldsymbol{u}_d \cdot \boldsymbol{x}= M^*_{d}. \end{aligned} \end{aligned}$$

Similar to the proof of Theorem 4.7, the associated Jacobian matrix to the above system is the modified Jacobian matrix $\tilde{J}$ defined in (25).

On the other hand, since $\boldsymbol{x}= \boldsymbol{x}^*$ is a non-degenerate equilibrium, Lemma 4.5 shows that

$$ \det (\tilde{J} ) \ne 0 \ \text { at } \ \boldsymbol{x}= \boldsymbol{x}^*. $$

The implicit function theorem shows that there exists an open interval containing $M_{1}$, that is,

$$ U = (M^{*}_{1} - \varepsilon , M^{*}_{1} + \varepsilon ), $$

such that there exists a unique and smooth family of equilibria

44 $$\begin{aligned} \boldsymbol{x}= (x_1 (M_{1}), \ldots , x_n (M_{1})), \end{aligned}$$

and it solves (43) for every $M_{1} \in U$. Taking the derivative with respect to $M_{1}$ on (43), together with (44), we obtain thatwhere $\prime$ indicates differentiation with respect to $M_{1}$. By Cramer’s rule, we compute

$$ x^{\prime }_n ( M_{1} ) = \frac{1}{\det (\tilde{J} ) } \det (\tilde{H}_{1}), $$

and thus conclude (42). $\square$

The following corollary is a direct consequence of Theorem 4.13.

#### Corollary 4.14

Consider the mass-action system $(G, \boldsymbol{k})$ which satisfies the assumptions in Theorem 4.13. Assume the system has a non-degenerate equilibrium $\boldsymbol{x}= \boldsymbol{x}^* \in \mathbb {R}^n_{>0}$ when $M = (M^*_{1}, \ldots , M^*_{d})$. Consider the following matrix:

$$ \tilde{H} = \begin{pmatrix} \frac{\partial f_{d+1}}{\partial x_1} & \cdots & \frac{\partial f_{d+1}}{\partial x_{n-1}} \\ \vdots & \ddots & \vdots \\ \frac{\partial f_{n}}{\partial x_1} & \cdots & \frac{\partial f_{n}}{\partial x_{n-1}} \end{pmatrix}. $$

Then $(G, \boldsymbol{k})$ exhibits complete infinitesimal concentration robustness at $(M^*_{1}, \ldots , M^*_{d})$ if

$$\begin{aligned} \{ \textbf{0} \} \subsetneq \ker { (\tilde{H}^{\intercal })} \ \text { at } \ \boldsymbol{x}= \boldsymbol{x}^*. \end{aligned}$$

#### Proof

Note that the matrix $\tilde{H}$ is obtained from the modified Jacobian matrix $\tilde{J}$ in (25) by deleting its first *d* rows and the last column. Suppose there exists a non-zero vector $\boldsymbol{v}\in \ker { (\tilde{H}^{\intercal })} \subset \mathbb {R}^{n-d}$ at $\boldsymbol{x}= \boldsymbol{x}^*$. By direct computation, for every $1 \le i \le d$,

$$ \big ( \underbrace{0, \ldots , 0,}_{d-1 \text { zeros}} \boldsymbol{v}^{\intercal } \big ) \tilde{H}_{i} = ( \underbrace{0, \ldots , 0}_{n-1 \text { zeros}} ) \ \text { at } \ \boldsymbol{x}= \boldsymbol{x}^*, $$

where $\tilde{H}_i$ is the $i^{th}$ modified homeostasis matrix defined in Definition 4.2. Therefore, we conclude this corollary from Theorem 4.13. $\square$

In addition, analogous to the infinitesimal homeostasis argument, the following results follow directly from Theorem 4.13 and the Deficiency Zero Theorem (Theorem 2.9).

#### Remark 4.15

Let $(G, \boldsymbol{k})$ be a complex-balanced system. Suppose $(G, \boldsymbol{k})$ satisfies (21)-(24) and the conservation laws are given by

$$\begin{aligned} \boldsymbol{u}_i \cdot \boldsymbol{x}(t) \equiv M_i \ \text { for } \ i = 1, \ldots , d. \end{aligned}$$

Assume the system has a complex-balanced equilibrium $\boldsymbol{x}= \boldsymbol{x}^*$ when $M = (M^*_{1}, \ldots , M^*_{d})$. Then for any $1 \le i \le d$, $(G, \boldsymbol{k})$ exhibits the $i^{th}$ infinitesimal concentration robustness at $M^*_{i}$ if and only if

$$\begin{aligned} \det ( \tilde{H}_{i} ) = 0 \ \text { at } \ \boldsymbol{x}= \boldsymbol{x}^*. \end{aligned}$$

Consequently, $(G, \boldsymbol{k})$ exhibits complete infinitesimal concentration robustness at $(M^*_{1}, \ldots , M^*_{d})$, if and only if the above holds for every $1 \le i \le d$. $\square$

#### Remark 4.16

Let *G* be a weakly reversible and deficiency zero E-graph, and let $(G, \boldsymbol{k})$ be a mass-action system in (20). Suppose $(G, \boldsymbol{k})$ satisfies (21)-(24) and the conservation laws are given by

$$\begin{aligned} \boldsymbol{u}_i \cdot \boldsymbol{x}(t) \equiv M_i \ \text { for } \ i = 1, \ldots , d. \end{aligned}$$

Assume the system has a positive equilibrium $\boldsymbol{x}= \boldsymbol{x}^*$ when $M = (M^*_{1}, \ldots , M^*_{d})$. Then for any $1 \le i \le d$, $(G, \boldsymbol{k})$ exhibits the $i^{th}$ infinitesimal concentration robustness at $M^*_{i}$ if and only if

$$\begin{aligned} \det ( \tilde{H}_{i} ) = 0 \ \text { at } \ \boldsymbol{x}= \boldsymbol{x}^*. \end{aligned}$$

Consequently, $(G, \boldsymbol{k})$ exhibits complete infinitesimal concentration robustness at $(M^*_{1}, \ldots , M^*_{d})$, if and only if the above holds for every $1 \le i \le d$. $\square$

#### Example 4.17

**Fig. 5:**
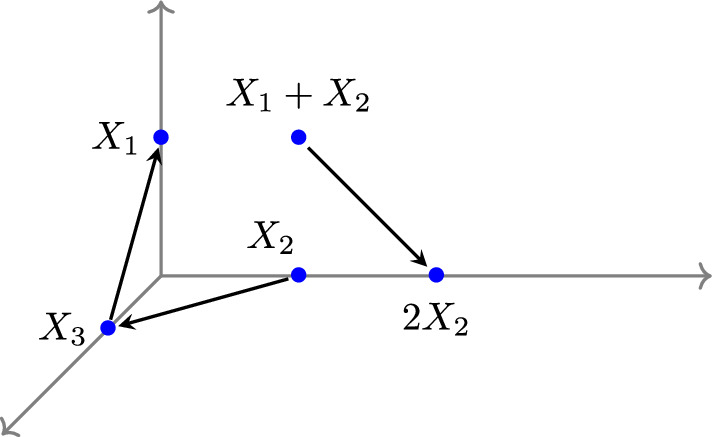
SIRS model reactions under mass-action kinetics

Consider the well-known epidemiological SIRS model, which extends the classic SIR (Susceptible-Infected-Recovered) framework by incorporating recovery and subsequent loss of immunity (Khalifi and Britton 2023), as follows:

$$\begin{aligned} X_1 + X_2 \xrightarrow {k_1} 2 X_2, \ \ X_2 \xrightarrow {k_2} X_3, \ \ X_3 \xrightarrow {k_3} X_1, \end{aligned}$$

where $X_1$, $X_2$, and $X_3$ represent the number of susceptible, infectious, and removed individuals (molecules), respectively. In addition, $k_1$ represents the rate of spread of the disease, $k_2^{-1}$ represents the duration of the disease, and $k_3^{-1}$ represents the duration of immunity.

Let $\boldsymbol{k}= (k_1, k_2, k_3) \in \mathbb {R}^3_{>0}$. We construct the E-graph *G* in Figure 5 and its associated mass-action system $(G, \boldsymbol{k})$ as follows::

45 $$\begin{aligned} \begin{aligned}&\frac{d x_1}{dt} = - k_1 x_1 x_2 + k_3 x_3, \\ &\frac{d x_2}{dt} = k_1 x_1 x_2 - k_2 x_2, \\ &\frac{d x_3}{dt} = k_2 x_2 - k_3 x_3. \end{aligned} \end{aligned}$$

We see that it admits the following conservation law:

46 $$\begin{aligned} x_1 (t) + x_2 (t) + x_3 (t) \equiv M_1. \end{aligned}$$

From (45) and (46), $(G, \boldsymbol{k})$ having an equilibrium $\boldsymbol{x}= \boldsymbol{x}^*$ when $M_1 = M^*_{1}$ is equivalent to solving the following system:

47 $$\begin{aligned} \frac{d x_1}{dt} = - k_1 x_1 x_2 + k_3 x_3 = 0, \ \ \frac{d x_2}{dt} = k_1 x_1 x_2 - k_2 x_2 = 0, \ \ x_1 (t) + x_2 (t) + x_3 (t) = M^*_{1}. \end{aligned}$$

We now consider the input-output system $(G, \boldsymbol{k}, {\mathcal {I}})$ with input ${\mathcal {I}}= M_1$ and output $x_3$.

(*a*) For $M_1 < \frac{k_2}{k_1}$, we compute that $\boldsymbol{x}^* = (M_1, 0, 0)$ is a non-degenerate and stable equilibrium of $(G, \boldsymbol{k})$. This kind of equilibrium is usually called a disease-free equilibrium since $x^*_2 = 0$. From (47), we obtain the modified Jacobian matrix given by

$$\begin{aligned} \tilde{J} = \begin{pmatrix} 1 & 1 & 1 \\ - k_1 x_2 & - k_1 x_1 & k_3 \\ k_1 x_2 & k_1 x_1 - k_2 & 0 \end{pmatrix}. \end{aligned}$$

Following Theorem 4.13, we compute the modified homeostasis matrix according to the conservation law (46) as follows:

$$\begin{aligned} H = \begin{pmatrix} - k_1 x_2 & - k_1 x_1 \\ k_1 x_2 & k_1 x_1 - k_2 \end{pmatrix}, \end{aligned}$$

and we derive that

$$ \det (H) = k_1 k_2 x^*_2 = 0 \ \text { at } \ { \boldsymbol{x}^* = (M_1, 0, 0). } $$

Therefore, we conclude that $(G, \boldsymbol{k}, {\mathcal {I}})$ exhibits complete infinitesimal concentration robustness at $\boldsymbol{x}^* = (M_1, 0, 0)$ when $M_1 < \frac{k_2}{k_1}$.

(*b*) For $M_1 > \frac{k_2}{k_1}$, we have $\boldsymbol{x}^* = (\frac{k_2}{k_1}, \frac{k_3}{k_2 + k_3} (M_1 - \frac{k_2}{k_1}), \frac{k_2}{k_2 + k_3} (M_1 - \frac{k_2}{k_1}))$ is a non-degenerate and stable equilibrium of $(G, \boldsymbol{k})$. This kind of equilibrium is usually called an endemic equilibrium, since $x^*_2 > 0$. Analogous to case (*a*), we obtain the modified homeostasis matrix as follows:

$$\begin{aligned} H = \begin{pmatrix} - k_1 x_2 & - k_1 x_1 \\ k_1 x_2 & k_1 x_1 - k_2 \end{pmatrix}, \end{aligned}$$

and thus

$$ \det (H) = k_1 k_2 x^*_2 \ne 0 \ \text { at } \ \boldsymbol{x}^* = (\frac{k_2}{k_1}, \frac{k_3}{k_2 + k_3} (M_1 - \frac{k_2}{k_1}), \frac{k_2}{k_2 + k_3} (M_1 - \frac{k_2}{k_1})). $$

Therefore, we conclude that $(G, \boldsymbol{k}, {\mathcal {I}})$ cannot exhibit infinitesimal concentration robustness at $\boldsymbol{x}^* = (\frac{k_2}{k_1}, \frac{k_3}{k_2 + k_3} (M_1 - \frac{k_2}{k_1}), \frac{k_2}{k_2 + k_3} (M_1 - \frac{k_2}{k_1}))$ when $M_1 > \frac{k_2}{k_1}$. $\square$

#### Remark 4.18

- From the form of the disease-free equilibrium $\boldsymbol{x}^* = (M_1, 0, 0)$, the third component (the output variable $X_3$) is always zero, indicating that the system exhibits not just infinitesimal, but absolute concentration robustness with respect to the input parameter $M_1 < \frac{k_2}{k_1}$.
- Similarly, from the form of the endemic equilibrium $\boldsymbol{x}^* = (\frac{k_2}{k_1}, \frac{k_3}{k_2 + k_3} (M_1 - \frac{k_2}{k_1}), \frac{k_2}{k_2 + k_3} (M_1 - \frac{k_2}{k_1}))$, the third component (the output variable $X_3$) is monotone with respect to the input parameter $M_1$. Therefore, it cannot exhibit infinitesimal concentration robustness at any $M_1 > \frac{k_2}{k_1}$.

#### Example 4.19

Let us consider the modified four-species Togashi-Kaneko (TK) network (for the original TK network, see Togashi and Kaneko (2001)), which is commonly used to study stochastic switching and autocatalytic interactions in chemical or biological systems. The reactions are given by

48 $$\begin{aligned} \begin{aligned} X_1 + X_2 \rightleftarrows 2 X_1, \ \ X_3 + X_4 \rightleftarrows 2 X_3, \ \ X_1 + X_3 \rightleftarrows X_2 + X_4. \end{aligned} \end{aligned}$$

Assuming all reaction rates equal 1, the associated mass-action system $(G, \boldsymbol{k})$ is

49 $$\begin{aligned} \begin{aligned}&\frac{d x_1}{d t} = x_1 x_2 - x_1^2 - x_1 x_3 + x_2 x_4, \\ &\frac{d x_2}{d t} = - x_1 x_2 + x_1^2 + x_1 x_3 - x_2 x_4, \\ &\frac{d x_3}{d t} = x_3 x_4 - x_3^2 - x_1 x_3 + x_2 x_4, \\ &\frac{d x_4}{d t} = - x_3 x_4 + x_3^2 + x_1 x_3 - x_2 x_4. \end{aligned} \end{aligned}$$

It admits two conservation laws:

$$\begin{aligned} \begin{aligned} x_1 + x_2 = M_1 \qquad x_3 + x_4 = M_2. \end{aligned} \end{aligned}$$

From (48) and (49), $(G, \boldsymbol{k})$ having an equilibrium $\boldsymbol{x}= \boldsymbol{x}^*$ when $(M_1, M_2) = (M^*_{1}, M^*_{2})$ is equivalent to solving the following system:

50 $$\begin{aligned} \begin{aligned}&\frac{d x_1}{dt} = x_1 x_2 - x_1^2 - x_1 x_3 + x_2 x_4 = 0, \quad x_1 + x_2 = M^*_{1}, \\ &\frac{d x_3}{d t} = x_3 x_4 - x_3^2 - x_1 x_3 + x_2 x_4 = 0, \quad x_3 + x_4 = M^*_{2}. \end{aligned} \end{aligned}$$

Fix $M_2 = 2$, we consider the input-output system $(G, \boldsymbol{k}, {\mathcal {I}})$ with input ${\mathcal {I}}= M_1$ and output $x_4$.

By computation, $\textbf{x}^* = (1, 1, 1, 1)$ is a non-degenerate equilibrium of $(G, \boldsymbol{k})$ when $M_1 = 2$. From (50), we obtain the modified Jacobian matrix given by

$$\begin{aligned} \tilde{J} = \begin{pmatrix} x_2 - 2 x_1 - x_3 & x_1 + x_4 & - x_1 & x_2 \\ 1 & 1 & 0 & 0 \\ - x_3 & x_4 & x_4 - 2 x_3 - x_1 & x_3 + x_2 \\ 0 & 0 & 1 & 1 \\ \end{pmatrix} \end{aligned}$$

Following Theorem 4.13, the modified homeostasis matrix is given by

$$\begin{aligned} H = \begin{pmatrix} x_2 - 2 x_1 - x_3 & x_1 + x_4 & - x_1 \\ - x_3 & x_4 & x_4 - 2 x_3 - x_1 \\ 0 & 0 & 1 \\ \end{pmatrix}, \end{aligned}$$

and we derive that

$$\begin{aligned} \det (H) = 0 \ \text { at } \boldsymbol{x}^* = (1, 1, 1, 1). \end{aligned}$$

Therefore, we conclude that $(G, \boldsymbol{k}, {\mathcal {I}})$ exhibits infinitesimal concentration robustness at $\boldsymbol{x}^* = (1, 1, 1, 1)$ when $M_1 = 2$. $\square$

We end this subsection with an interesting result on infinitesimal homeostasis and infinitesimal concentration robustness when an E-graph consists of one reversible pair of reactions involving two species.

#### Corollary 4.20

Consider the following mass-action system $(G, \boldsymbol{k})$:which admits the conservation law given by:

$$\begin{aligned} (b-d) x_1 (t) + (c-a) x_2 (t) \equiv M_1. \end{aligned}$$

Assume the system has a positive equilibrium $\boldsymbol{x}= (x^*_1, x^*_2)$ when $\boldsymbol{k}= (k^*_1, k^*_2)$ and $M_1 = M^*_1$. Consider the associated input-output system $(G, \boldsymbol{k}, {\mathcal {I}})$ with the output parameter $x_2$.

- If the input parameter ${\mathcal {I}}= k_i$ with $i = 1, 2$, infinitesimal homeostasis occurs at $k^*_{i}$ when $b = d$.
- If the input parameter ${\mathcal {I}}= M_1$, complete infinitesimal concentration robustness occurs at $M^*_{1}$ when $a = c$.

#### Proof

Under the mass-action kinetic, the system $(G, \boldsymbol{k})$ follows

51 $$\begin{aligned} \begin{aligned}&\frac{ d x_1}{ dt } = k_1 (x_1)^a (x_2)^b (c-a) + k_2 (x_1)^c (x_2)^d (a-c), \\ &\frac{ d x_2}{ dt } = k_1 (x_1)^a (x_2)^b (d-b) + k_2 (x_1)^c (x_2)^d (b-d), \end{aligned} \end{aligned}$$

and it admits the following conservation law:

52 $$\begin{aligned} (d-b) \frac{ d x_1}{ dt } - (c-a) \frac{ d x_2}{ dt } = 0. \end{aligned}$$

We exclude the case when both $a = c$ and $b = d$ hold because we always assume no self-loops in the E-graph. Note that the E-graph *G* is weakly reversible and deficiency zero. The Deficiency Zero Theorem shows that $\boldsymbol{x}= (x^*_1, x^*_2)$ is a complex-balanced equilibrium.

We first consider the input parameter ${\mathcal {I}}= k_i$ with $i = 1, 2$. Without loss of generality, we assume ${\mathcal {I}}= k_1$ and $a \ne c$. Note that the system (51), together with the conservation law (52), is equivalent to the following system:

53 $$\begin{aligned} \begin{aligned}&\frac{ d x_1}{ dt } = f_1 (x_1, x_2) = k_1 (x_1)^a (x_2)^b (c-a) + k_2 (x_1)^c (x_2)^d (a-c), \\ &(d-b) x_1 - (c-a) x_2 = M_1, \end{aligned} \end{aligned}$$

whose Jacobian matrix is the modified Jacobian matrix of (51) and (52).

Since the equilibrium $\boldsymbol{x}= (x^*_1, x^*_2)$ with $\boldsymbol{k}= (k^*_1, k^*_2)$ and $M_1 = M^*_1$ is complex-balanced, we have shown that the corresponding Jacobian matrix of (53) is non-singular, that is,

$$ \det \begin{pmatrix} d-b & a-c \\ \frac{\partial f_1}{\partial x_1} & \frac{\partial f_1}{\partial x_2} \end{pmatrix} \ne 0 \ \text { at } \ \boldsymbol{x}= (x^*_1, x^*_2) \ \text { with } \ \boldsymbol{k}= (k^*_1, k^*_2), \ M_1 = M^*_1. $$

Using the Cramer’s rule, we obtain $x_2^{\prime } (k^*_{1}) = 0$ if

$$ \det \begin{pmatrix} d-b & 0 \\ \frac{\partial f_1}{\partial x_1} & (x_1)^a (x_2)^b (a-c) \end{pmatrix} = 0. $$

Therefore, infinitesimal homeostasis occurs at $k^*_{1}$ when $b = d$. Analogously, the same conclusion holds if the input parameter ${\mathcal {I}}= k_2$.

Part (*b*) follows similarly to part (*a*) by considering the input parameter ${\mathcal {I}}= M_1$. $\square$

### Stochastic systems

In this section, we consider infinitesimal concentration robustness in stochastic reaction networks. We start by introducing *stochastic mass-action systems*. Our exposition largely follows Anderson and Kurtz (2015).

#### Definition 4.21

Let $(G, \boldsymbol{k})$ be a mass-action system as defined in (20). The **associated stochastic mass-action system** generated by $(G, \boldsymbol{k})$ is

54 $$\begin{aligned} \boldsymbol{X}(t) = \boldsymbol{x}(0) + \sum _{\boldsymbol{y}\rightarrow \boldsymbol{y}' \in E} Y_{\boldsymbol{y}\rightarrow \boldsymbol{y}'} \big ( \int ^{t}_{0} \lambda _{\boldsymbol{y}\rightarrow \boldsymbol{y}'} ( \boldsymbol{X}(s)) d s \big ) (\boldsymbol{y}' - \boldsymbol{y}), \end{aligned}$$

where $\boldsymbol{x}(0)$ is the deterministic initial condition, $\boldsymbol{X}(t) \in \mathbb {Z}_{>0}^n$ is a vector of random variables at time $t > 0$, and $\{ Y_{\boldsymbol{y}\rightarrow \boldsymbol{y}'} \}$ are independent unit rate Poisson processes with the intensity

$$\begin{aligned} \lambda _{\boldsymbol{y}\rightarrow \boldsymbol{y}'} ( \boldsymbol{x}(s) ) = k_{\boldsymbol{y}\rightarrow \boldsymbol{y}'} \prod ^{n}_{i = 1} \frac{x_i !}{(x_i - y_{i}) !}. \end{aligned}$$

Denote the closed, irreducible components of the state space of a countable Markov chain by $\{\Gamma \}$. Then all stationary distributions of the chain can be written as

55 $$\begin{aligned} \pi = \sum _{\Gamma } \alpha _{\Gamma } \pi _{\Gamma }, \end{aligned}$$

where $\alpha _{\Gamma } \ge 0$, $\sum _{\Gamma } \alpha _{\Gamma } = 1$, and where $\pi _{\Gamma }$ is the unique stationary distribution satisfying $\pi _{\Gamma }(\Gamma ) = 1$, for those $\Gamma$ for which a stationary distribution exists. In Anderson and Kurtz (2015), it is shown that when $(G, \boldsymbol{k})$ is a complex-balanced system satisfying (21)-(24) and $\boldsymbol{x}^*$ is a complex-balanced equilibrium in (20), then the associated stochastic mass-action system defined in (54) admits a stationary distribution (55) where $\pi _\Gamma$’s are in product-form

$$\begin{aligned} \pi _\Gamma (\boldsymbol{x}) = C_\Gamma \prod ^{n}_{i = 1} \frac{(x^*_i)^{x_i}}{x_i !} \quad \text {for} \quad \boldsymbol{x}\in \Gamma , \end{aligned}$$

and zero otherwise with $C_\Gamma$ being a normalizing constant. Furthermore, from (24) and (54) the expectation of $\boldsymbol{X}(t)$ satisfies

$$\begin{aligned} \boldsymbol{u}_i \cdot E (\boldsymbol{X}(t)) \equiv M_i \ \text { for } \ i = 1, \ldots , d \end{aligned}$$

where $M_i = \boldsymbol{u}_i \cdot \boldsymbol{x}(0)$. This follows in view of the definition of $\boldsymbol{u}_i$ in (23) implying $\boldsymbol{u}_i \cdot (\boldsymbol{y}' - \boldsymbol{y})=0$ in (54). Let the input parameter be the conservation law constant $M_i$, and let the output parameter be the expectation of the stationary distribution of the species $X_n$ (denoted by $E (X_n)$ or $E_n$). This allows us to define the notion of *infinitesimal concentration robustness* in stochastic mass-action systems as follows.

#### Definition 4.22

Let $(G, \boldsymbol{k})$ be a complex-balanced system that satisfies (21)-(24). Consider the associated stochastic mass-action system with the distribution of $\boldsymbol{X}(t)$ satisfying

56 $$\begin{aligned} \boldsymbol{u}_i \cdot E (\boldsymbol{X}(t)) \equiv M_i \ \text { for } \ i = 1, \ldots , d. \end{aligned}$$

Then $\boldsymbol{X}(t)$ admits a stationary distribution and we denote by $E_n$ the expectation of the stationary distribution of the species $x_n$. For any $1 \le i \le d$, suppose the input-output function $E_n (M_{i})$ is well-defined in a neighborhood of $M^*_{i}$. We say that the $i^{th}$ **infinitesimal concentration robustness** occurs at $M^*_{i}$ if

57 $$\begin{aligned} \frac{d}{d M_{i}} E_n (M^*_{i}) = 0. \end{aligned}$$

Moreover, suppose the input-output function $E_n (M_{i})$ is well-defined in a neighborhood of $M^*_{i}$ for every $1 \le i \le d$. The **complete infinitesimal concentration robustness** occurs at $M^* = (M^*_{1}, \ldots , M^*_{d})$, if the $i^{th}$ infinitesimal concentration robustness occurs at $M^*_{i}$ for every $1 \le i \le d$.

#### Remark 4.23

Note that in (57) above, we do not need to specify the stationary distribution of the form (55) but merely its expectation. In particular, the input-output relation remains valid if we fix a single reference distribution (e.g., corresponding to a fixed complex-balanced equilibrium) and consider how its marginal expectation varies as the input parameters $M_i$ change. This expectation aligns with the deterministic equilibrium, and the robustness condition simply quantifies the insensitivity to infinitesimal perturbations in the (deterministic) inputs.

#### Definition 4.24

((Anderson and Kurtz (2015), Definition 2.8))

An E-graph *G* is termed a **first-order E-graph** if all reactions are of the form

$$ \emptyset \rightarrow * \ \text { or } \ X_i \rightarrow * $$

where $*$ represents any linear combination of the species.

#### Lemma 4.25

(Anderson and Kurtz (2015))

Let *G* be a first-order E-graph, and let $(G, \boldsymbol{k})$ be a complex-balanced system that satisfies (21)-(24) and the conservation laws are given by

$$\begin{aligned} \boldsymbol{u}_i \cdot \boldsymbol{x}(t) \equiv M_i \ \text { for } \ i = 1, \ldots , d, \end{aligned}$$

where $\boldsymbol{x}(t) =E(\boldsymbol{X}(t))$. Suppose $\boldsymbol{x}^*$ is the positive equilibrium for $(G, \boldsymbol{k})$ when $M = M^*$, then the associated stochastic mass-action system admits a stationary distribution $\pi ^*$ whose expectation satisfies

$$\begin{aligned} E_{\pi ^*} \boldsymbol{X}= \boldsymbol{x}^*. \end{aligned}$$

#### Proof

For completeness, we sketch the proof of this lemma adapted from Section 2.2 of Anderson and Kurtz (2015). Since *G* is first-order, from Definition 4.24 there are only two types of reactions. First, when the reaction is $\emptyset \xrightarrow []{k} *$, the associated intensity function is the reaction rate constant, that is,

$$ \lambda _{\emptyset \rightarrow *} = k. $$

Second, when the reaction is $X_i \xrightarrow []{k} *$, the associated intensity function is

$$ \lambda _{X_i \rightarrow *} = k x_i. $$

Hence, for every reaction $\boldsymbol{y}\rightarrow \boldsymbol{y}'$ belonging to the above two cases, we obtain

$$ E [\lambda _{\boldsymbol{y}\rightarrow \boldsymbol{y}'} ( \boldsymbol{X}(s))] = \lambda _{\boldsymbol{y}\rightarrow \boldsymbol{y}'} (E [ \boldsymbol{X}(s)]) = \lambda _{\boldsymbol{y}\rightarrow \boldsymbol{y}'} ( \boldsymbol{x}(s)) \ \text { for any } s > 0. $$

By (54), this implies that $E (\boldsymbol{X}(t)) =\boldsymbol{x}(t)$ is a solution to the complex-balanced system $(G, \boldsymbol{k})$. Together with the assumed conservation law, this implies that in the stochastic system we have

$$\begin{aligned} \boldsymbol{u}_i \cdot \boldsymbol{x}(t) \equiv M^*_i \ \text { for } \ i = 1, \ldots , d, \end{aligned}$$

and the lemma’s assertion follows. $\square$

#### Theorem 4.26

Let *G* be a first-order E-graph, and let $(G, \boldsymbol{k})$ be a complex-balanced system that satisfies (21)-(24) and the conservation laws are given by

$$\begin{aligned} \boldsymbol{u}_i \cdot \boldsymbol{x}(t) \equiv M_i \ \text { for } \ i = 1, \ldots , d, \end{aligned}$$

where $\boldsymbol{x}(t) =E(\boldsymbol{X}(t))$. Suppose $\boldsymbol{x}^*$ is the positive equilibrium for $(G, \boldsymbol{k})$ when $M = M^*$. Consider the associated stochastic mass-action system. Then for any $1 \le i \le d$, the system exhibits the $i^{th}$ infinitesimal concentration robustness at $M^*_{i}$ if and only if

$$\begin{aligned} \det ( \tilde{H}_{i} ) = 0 \ \text { at } \ \boldsymbol{x}= \boldsymbol{x}^*. \end{aligned}$$

Furthermore, the system exhibits complete infinitesimal concentration robustness at $(M^*_{1}, \ldots , M^*_{d})$, if and only if the above holds for every $1 \le i \le d$.

#### Proof

From Lemma 4.25, for $M = M^*$ the stationary solution $\pi ^*$ satisfies

$$\begin{aligned} E_{\pi ^*}\boldsymbol{X}= \boldsymbol{x}^*. \end{aligned}$$

Recall Theorem 2.7, $\boldsymbol{x}^*$ is a complex-balanced equilibrium that is linearly stable in each stoichiometric compatibility class. The assertion of the theorem holds thus by Theorem 4.13. $\square$

#### Remark 4.27

In the context of the above theorem, while the complex-balance guarantees the existence of a product-form stationary distribution and facilitates the analysis, it is not strictly required for the infinitesimal robustness results. As shown in Lemma 4.25, for first-order systems with affine intensities, the expectations evolve according to deterministic dynamics. Consequently, similar conclusions may hold even without the complex-balanced assumption, provided that stationary distributions exist.

#### Corollary 4.28

Let *G* be a first-order E-graph, and let $(G, \boldsymbol{k})$ be a complex-balanced system that satisfies (21)-(24) and the conservation laws are given by

$$\begin{aligned} \boldsymbol{u}_i \cdot \boldsymbol{x}(t) \equiv M_i \ \text { for } \ i = 1, \ldots , d. \end{aligned}$$

Suppose $(G, \boldsymbol{k})$ admits a positive equilibrium when $M = M^*$.

- For any $1 \le i \le d$, assume the associated stochastic mass-action system exhibits the $i^{th}$ infinitesimal concentration robustness at $M^*_{i}$. Then it exhibits the $i^{th}$ infinitesimal concentration robustness at every $\widetilde{M}_{i}$, where $(G, \boldsymbol{k})$ admits a positive equilibrium when $M = \widetilde{M}$.
- If the associated stochastic mass-action system exhibits complete infinitesimal concentration robustness at $M^*$, then it exhibits complete infinitesimal concentration robustness at every $\widetilde{M}$, where $(G, \boldsymbol{k})$ admits a positive equilibrium when $M = \widetilde{M}$.

#### Proof

From Theorem 4.26, the stochastic system exhibiting the $i^{th}$ infinitesimal concentration robustness at $M^*_{i}$ shows that

$$\begin{aligned} \det ( \tilde{H}_{i} ) = 0 \ \text { at } \ \boldsymbol{x}= \boldsymbol{x}^*. \end{aligned}$$

Since *G* is a first-order E-graph, the Jacobian matrix is fixed under any equilibrium. Given any conservation law $M = \widetilde{M}$, $(G, \boldsymbol{k})$ admits a unique complex-balanced equilibrium $\tilde{\boldsymbol{x}}$. Therefore,

$$\begin{aligned} \det ( \tilde{H_i} ) = 0 \ \text { at } \ \boldsymbol{x}= \tilde{\boldsymbol{x}}. \end{aligned}$$

and thus we conclude it exhibits the $i^{th}$ infinitesimal concentration robustness at every $\widetilde{M}_{i}$. We omit the proof of part (*b*) as it is very similar to part (*a*). $\square$

## Discussion

In this paper, we have derived some methods that allow us to establish the properties of infinitesimal homeostasis and infinitesimal concentration robustness for reaction networks under the mass-action law in both deterministic and stochastic settings. Our results provide an extension of several earlier ones, particularly those discussed in Golubitsky and Stewart (2017); Craciun and Deshpande (2022).

Our work provides several new ways of expanding the theory of homeostasis to reaction network systems, leading to new opportunities for applications. In particular, the important expansion of the classical theory of homeostasis is achieved by means of the analysis of the modified Jacobian matrix in systems with conservation laws (see Sect. 4.1). As we pointed out in Example 4.17, this method applies for instance the SIR-type models due to their important applications in epidemiology. By studying the modified Jacobian matrix introduced in Sect. 4.1, we can analyze SIR and similar models and compute infinitesimal concentration robustness without relying on an E-graph representation of a reaction network.

While beyond the scope of our current work, the approach proposed here holds potential for application to reaction networks with multiple input parameters. For instance, a reaction rate constant and a conservation law constant could be jointly used as input parameters. Therefore, it is of interest to determine under what conditions the chosen output variable can exhibit infinitesimal homeostasis with respect to multiple inputs. Some relevant ideas have been recently proposed in this area by Golubitsky and Stewart (2018); Madeira and Antoneli (2023a, 2023b). Besides multiple parameter inputs, there are also other intriguing generalizations of the notion of homeostasis in reaction networks. The first one is inspired by bifurcation theory. As discussed in this paper, the infinitesimal homeostasis occurs at ${\mathcal {I}}_0$ if $x_n'({\mathcal {I}}_0) = 0$, where ${\mathcal {I}}\mapsto x_n({\mathcal {I}})$ is the input-output function. Furthermore, if $x_n'({\mathcal {I}}_0) = x_n''({\mathcal {I}}_0) = 0$ and $x_n'''({\mathcal {I}}_0) \ne 0$, then the output variable exhibits *chair homeostasis* at ${\mathcal {I}}_0$. The authors in Nijhout et al. (2014) associated homeostasis with “chairs”, referring to curves that are strictly monotonic except for a flat segment. An infinitesimal characterization of a *chair point* was introduced in Golubitsky and Stewart (2017, 2018). Chair homeostasis reflects a stronger form of robustness, in which the input-output response remains flatter than typically expected. It is therefore worthwhile to study chair homeostasis in reaction networks both without and under conservation laws. In this context, it appears that our two theorems from Sect. 4 can also be extended to study the chair homeostasis.

The second area for potential future expansion is the investigation of homeostasis in general complex-balanced stochastic systems. Although we presented some basic results on such systems in Sect. 4.3, these were limited to stochastic reaction networks with first-order E-graphs. Extending this to networks with source complexes consisting of more than one species (e.g., SIR models) appears feasible. Since it is crucial to understand how infinitesimal homeostasis occurs in general networks, we hope to be able to pursue such work in the future.

## Footnotes

1 Note that when $\boldsymbol{y}= \emptyset$, we have $\boldsymbol{x}^{\boldsymbol{y}} = x_1^{0} \ldots x_n^{0}$ = 1.

2 An equilibrium point is called *hyperbolic* if the Jacobian matrix evaluated at that point has no eigenvalues with zero real parts. It is called *non-degenerate* if the Jacobian matrix is non-singular at that point.
